# Clusterin drives myeloid bias in aged hematopoietic stem cells by regulating mitochondrial function

**DOI:** 10.1038/s43587-025-00908-z

**Published:** 2025-06-30

**Authors:** Ninghe Sun, Chun-Hsin Lin, Michelle Y. Li, Yuting Wang, Danyang Chen, Xiangle Ren, Feng Zhang, Yi Zhang

**Affiliations:** 1 Program in Cellular and Molecular Medicine, Boston Children’s Hospital, Boston, MA USA; 2 Division of Hematology/Oncology, Department of Pediatrics, Boston Children’s Hospital, Boston, MA USA; 3 Howard Hughes Medical Institute, Boston Children’s Hospital, Boston, MA USA; 4 Department of Cardiology, Boston Children’s Hospital, Boston, MA USA; 5 Broad Institute of Massachusetts Institute of Technology and Harvard, Cambridge, MA USA; 6 Department of Genetics, Harvard Medical School, Boston, MA USA; 7 Harvard Stem Cell Institute, Boston, MA USA

**Keywords:** Ageing, Ageing

## Abstract

Aged hematopoietic stem cells (HSCs) exhibit diminished self-renewal and myeloid-biased differentiation with a decline in hematopoiesis and adaptive immune function. However, the molecular regulation of this impaired function remains largely unknown. Here, through an in vivo CRISPR–Cas9-based screen, we uncovered clusterin (Clu) as a driver of biased differentiation. Clu is upregulated in aged HSCs, and its knockout diminishes biased differentiation. Clu promotes mitochondrial hyperfusion by interacting with Mfn2 in aged HSCs, and its ablation attenuates oxidative phosphorylation, improves mitophagy, and reverses myeloid-biased differentiation via the OXPHOS-p38-Cebpb axis. Transplantation of Clu-depleted aged HSCs into middle-aged mice results in balanced hematopoiesis and improved physical functions. Together, our data identify Clu as a critical regulator of aging-associated myeloid bias and reveal an Mfn2-OXPHOS-p38-Cebpb axis as the mechanism underlying how Clu upregulation in aged HSCs leads to myeloid-biased differentiation, providing a target for rejuvenation of aged hematopoietic and immune systems.

## Abstract (web summary)

Performing a CRISPR–Cas9 screen in aged mice, Sun and colleagues identify clusterin as a driver of myeloid-biased hematopoietic stem cell differentiation in aging, through regulation of mitochondrial function via a Mnf2-OXPHOS-p38-Cebpb regulatory axis.

## Main

The increased lifespan has resulted in a global increase in the aged population, prompting great interest in healthy aging research. Aging is a natural process accompanied by functional decline in tissues and organs, serving as a critical risk factor for chronic diseases and human pathologies^1^. Hematopoietic stem cells (HSCs) are the cell source of the hematopoietic and immune systems. Thus, maintaining their life-long self-renewal and differentiation capacity is critical. However, aging causes genetic and epigenetic changes that result in HSC dysfunction^2^. Due to aging-related dysfunctional hematopoiesis, the incidence of hematologic malignancies is elevated among older adults^3^. Interestingly, transfusion of young blood into an aged mouse rejuvenates multiple organs^4^, indicating that the aged hematopoietic system contributes to age-related whole-body functional decline.

As the source of all cell types of the hematopoietic system, long-term HSCs (LT-HSCs, referred to as HSCs) have been found to exhibit major malfunctions during aging. In the most undifferentiated form, HSCs can be quiescent, self-renew or differentiate into specialized blood cells under the influence of extrinsic factors such as cytokines and intrinsic factors such as transcription factors^5^. During aging, dysregulation of these factors leads to altered HSC fate and disrupts the HSC differentiation balance^6^. Furthermore, replacing HSCs of old mice with those of young mice can extend the lifespan of the old recipient mice^7^, suggesting that HSCs play a central role in systematic aging.

The hallmarks of aged HSCs include reduced self-renewal capacity, reduced homing efficiency and myeloid-biased differentiation^2^. A previous study showed that a subset of HSCs exhibit decreased lymphoid differentiation capacity during aging in transplanted mice, leading to a shift toward myeloid differentiation^8^. This myeloid-biased subset of HSCs proliferates more than balanced HSCs in aged mice with an overproduction of myeloid cells, resulting in a compromised immune system^9^. This finding indicates that myeloid-biased differentiation of aged HSCs is one of the main defects contributing to aging-related functional decline. However, the key players that cause the aging-associated myeloid bias and the underlying mechanisms are unclear, preventing the development of intervention strategies. Therefore, understanding the mechanisms underlying aging-associated myeloid-biased differentiation is an urgent need.

Recent studies suggest that mitochondria dysfunction is a prominent mechanism of aging-associated functional decline of HSCs^10^. For example, mitochondrial membrane potential is a determinant of the age-associated transcription state of HSCs affecting transcriptional rate, myeloid bias, reactive oxygen species (ROS) levels, and DNA repair^11^. Additionally, autophagy is critical for maintaining HSC stemness and regenerative potential by regulating mitochondria clearance^12^ while also playing a role in maintaining HSC quiescence in response to chronic inflammation and glycolytic impairment during aging^13^. By inducing mitochondrial recycling, urolithin A-supplemented food restores lymphoid compartments, boosts HSC function, and improves immune responses against viral infection in old mice^14^. Nicotinamide riboside administration also restores metabolism to a youthful state by promoting NAD homeostasis and clearance of defective mitochondria^15^. Collectively, these studies demonstrate that HSC state is directly linked to mitochondrial function in the context of aging.

Here, by performing a CRISPR-based loss-of-function screen of consistently upregulated genes in aged murine HSCs, we revealed clusterin (Clu) as a top candidate responsible for driving myeloid-biased differentiation in aged HSCs, which was confirmed by both loss- and gain-of-function studies. Mechanistically, we showed that an age-dependent increase in *Clu* expression leads to excessive mitochondrial fusion and oxidative phosphorylation (OXPHOS) with an increase in ROS, which activates the p38 MAPK signaling pathway, resulting in increased *Cebpb* expression. As a key transcription factor driving myeloid cell differentiation, upregulation of *Cebpb* leads to myeloid-biased differentiation of aged HSCs. Depletion of Clu can reverse the biased differentiation and alleviate aging-associated functional decline. Thus, our study provides insights into the mechanisms of myeloid-biased differentiation in aged HSCs and suggests manipulation of the Mfn2-OXPHOS-p38-Cebpb axis as a promising strategy for rejuvenation.

## Results

### Identifying Clu as a regulator of myeloid bias in aged HSCs

**Extended Data Fig. 1:**
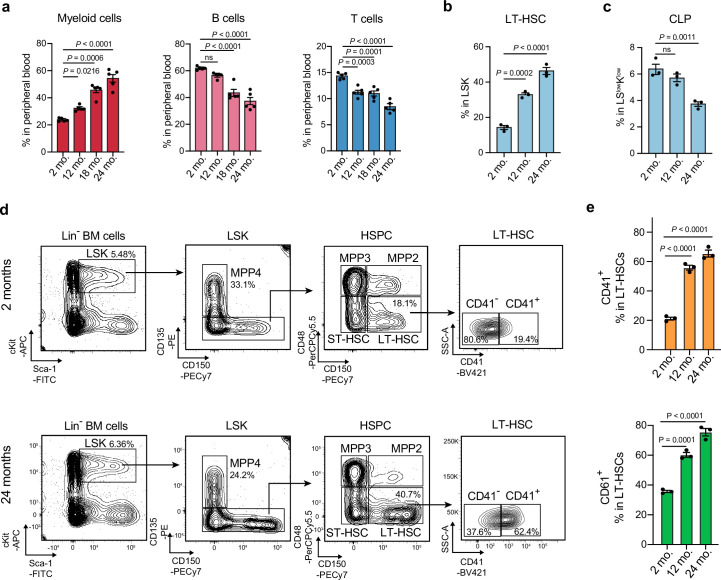
Myeloid-biased hematopoiesis in aged mice. (**a**) Frequency of myeloid, B, and T cells in peripheral blood (PB) from mice at 2, 12, 18, and 24 months (n = 5 per group). (**b**) Percentage of LT-HSCs in LSK (Lin^–^Sca1^+^cKit^+^) cells from mice at 2, 12, and 24 months (n = 3 per /group). (**c**) Percentage of common lymphoid progenitors (CLP) in LS^low^K^low^ (Lin^–^Sca1^low^cKit^low^) cells from mice at 2, 12, and 24 months (n = 3 per group). (**d**, **e**) Representative FACS plots (**d**) and quantification (**e**) of CD41 or CD61 positive cells within the LT-HSCs in 2, 12, 24-month-old mice (n = 3 per group). One-way ANOVA with Dunnett’s multiple comparison test (**a**–**c**, **e**). Error bars represent mean ± SEM (**a**–**c**, **e**). Source data

HSCs gradually lose their regenerative potential and produce a myeloid-biased output during aging, resulting in poor immune responses to infections^16^. Consistent with previous reports^17,18^, we observed increased frequency of myeloid cells relative to lymphoid cells (B and T cells) (Extended Data Fig. 1a), increased frequency of LT-HSCs (Extended Data Fig. 1b), reduced frequency of common lymphoid progenitors (Extended Data Fig. 1c) and increased frequency of CD41^+^ or CD61^+^ old HSCs (oHSCs) (Extended Data Fig. 1d,e) during aging.

**Fig. 1:**
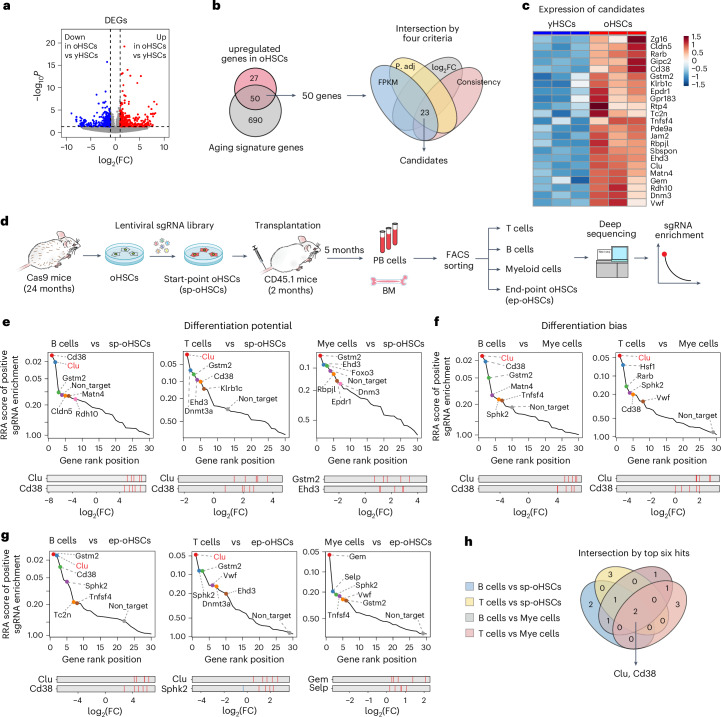
CRISPR screen identified Clu and Cd38 as regulators of myeloid-biased hematopoiesis. **a**, Volcano plot showing differentially expressed genes (DEGs) of oHSCs from old mice (24 months) compared with yHSC from young mice (2 months) (*n* = 3 per group; two-sided Wald test *P* value). **b**, Intersection of upregulated genes in oHSCs from our dataset and the public HSC aging signature genes (left panel). The four criteria used for inclusion (right panel). FPKM, fragments per kilobase of transcript per million mapped reads; log_2_FC, log_2_ fold change; P. adj, adjusted *P* value. **c**, Heatmap of the expression of candidates included in the CRISPR loss-of-function screen. **d**, Workflow of in vivo CRISPR screen in oHSCs. **e**, Rank plot showing screening results comparing mature B, T, and myeloid cells with sp-oHSCs. The *x* axis indicates the rank order of genes. The *y* axis indicates the gene’s robust rank aggregation (RRA) score for positive enrichment of sgRNAs. Bottom panel shows the distribution of the average fold change of three sgRNAs with the five barcodes for the top hits. Red bars indicate sgRNAs enrichment in B, T, or myeloid cells compared with sp-oHSCs for the top hits. **f**, Rank plot showing screening results comparing mature B or T cells with myeloid cells. The axes are the same as those in **e**. Red bars indicate sgRNAs enrichment in B or T cells compared with myeloid cells for the top hits. **g**, Rank plot showing screening results comparing mature B, T, and myeloid cells with ep-oHSCs. The axes and red bars are the same as those in **e**. Red bars indicate sgRNAs enrichment in B or T cells compared with ep-oHSCs for the top hits. **h**, Four pairwise comparisons identified *Clu* and *Cd38* as the top hits. PB, peripheral blood; BM, bone marrow; Mye, myeloid. Source data

To understand how the aging process leads to the functional decline of HSCs, we compared the transcriptomes of oHSCs with young HSCs (yHSCs) and identified 77 significantly upregulated genes in oHSCs (FPKM > 1, adjusted *P* value < 0.05, log_2_FC > 0.5) (Fig. 1a,b). We narrowed down this gene list to 50 by overlapping them with a previously identified 740 aging signature gene list^19^ (Fig. 1b and Supplementary Table 1). We ranked these 50 genes based on four criteria: (1) expression of candidate based on FPKM, (2) adjusted *P* value, (3) fold changes and (4) consistency in the aging signature^19^, resulting in 23 top candidates for in vivo CRISPR-based loss-of-function screen (Fig. 1c). We also included six genes (*Foxo3*, *Sphk2*, *Selp*, *Dnmt3a*, *Hsf1* and *Sirt2*) with known functions in hematopoiesis to explore their potential roles in regulating differentiation of aged HSCs.

To identify the genes involved in regulating hematopoiesis in aged HSCs, we used an in vivo barcode-based CRISPR screening method^20^. We performed CRISPR gene editing in oHSCs isolated from aged Cas9 mice (24 months). To target the candidate genes, lentiviral libraries of sgRNAs (three sgRNAs/gene) with five different barcodes for each sgRNA were constructed. Cas9-expressing oHSCs were infected with the libraries (referred to as start-point oHSCs (sp-oHSCs)). Then the sp-oHSCs were transplanted into lethally irradiated CD45.1 recipient mice. After 5 months, peripheral blood and bone marrow from the recipient mice were collected and hematopoietic cell types (CD11b^+^ myeloid cells, CD3^+^ T cells, B220^+^ B cells and LSK CD48^−^ CD150^+^ HSCs, hereafter referred to as endpoint oHSCs (ep-oHSCs)) were sorted for sgRNA sequencing and enrichment analysis (Fig. 1d).

Our screen system enabled three pairwise comparisons of HSC-derived immune cells to reveal loss-of-function effects of these genes on the lineage commitment from sp-oHSCs to mature B cells, T cells and myeloid cells (referred to as differentiation potential). Analysis of the results uncovered key roles of several genes in regulating HSC differentiation to specific hematopoietic lineages. For example, *Cd38* and *Clu* knockouts (KOs) strongly promote differentiation to B cells and T cells (Fig. 1e, left and middle panels), whereas *Gstm2* and *Foxo3* KOs strongly promote myeloid differentiation (Fig. 1e, right panel). Consistent with previous demonstration that *Foxo3* KO results in myeloproliferative syndrome^21^, our screen identified *Foxo3* as a top hit for myeloid differentiation after *Foxo3* KO (Fig. 1e, right panel). Importantly, our screen approach allowed a direct pairwise comparison between myeloid and lymphoid lineages to reveal genes whose loss of function resulted in differentiation bias of aged HSCs. The results revealed *Clu*, *Cd38*, *Gstm2* and *Hsf1* as the top hits (Fig. 1f). We also compared the B, T, and myeloid cells with ep-oHSCs, which reflect the differentiation of ep-oHSC to mature immune cells, and revealed *Clu*, *Gstm2*, *Cd38,* and *Sphk2* as the top hits (Fig. 1g). Inhibition of *Sphk2* sustains long-term self-renewal and increases regenerative potential^22^. Consistently, our screen also indicated that loss of *Sphk2* promotes oHSC differentiation (Fig. 1g). Finally, four pairwise comparisons identified *Clu* and *Cd38* as the common genes whose loss of function resulted in increased lymphoid differentiation (Fig. 1h).

### Manipulation of Clu levels affects HSC differentiation

**Extended Data Fig. 2:**
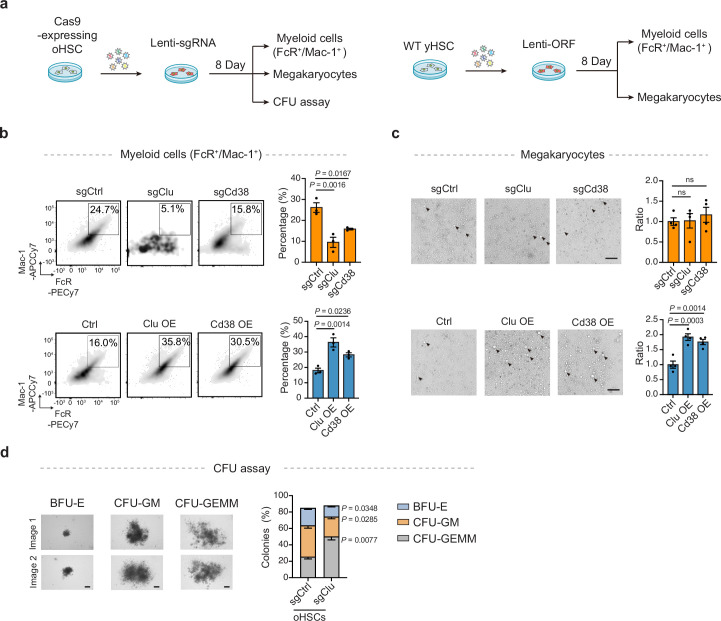
Manipulation of *Clu* or *Cd38* levels in HSCs affect differentiation of HSCs *in vitro.* (**a**) Workflow for *in vitro* verification of the role of *Clu* and *Cd38* in regulating HSC differentiation. (**b**) Representative FACS plots (left) and quantification (right) of FcR^+^/Mac-1^+^ myeloid cells after in vitro differentiation culture (n = 3 per group). (**c**) Representative images (left) and quantification (right) of megakaryocyte ratio after in vitro differentiation culture (n = 4 per group). Scale bar, 100 μm. (**d**) Colony-forming unit (CFU) assay in control and *Clu* KO oHSCs. Left panels display colony morphologies of burst-forming unit-erythroid (BFU-E); colony-forming unit-granulocyte, macrophage (CFU-GM); and colony-forming unit-granulocyte, erythrocyte, macrophage, megakaryocyte (CFU-GEMM). Right panel shows the colony type distribution in percentage of the colonies (n = 4 per group). Scale bar, 200 μm. One-way ANOVA with Dunnett’s multiple comparison test (**b**, **c**). Unpaired two-tailed Student’s t-test (**d**). Error bars represent mean ± SEM (**b**–**d**). Ns: no significant difference. Source data

As an enzyme responsible for NAD^+^ metabolism, the role of Cd38 in aging has been well documented^23^. Thus, our study focuses on Clu but uses Cd38 as a control whenever appropriate. Clu was reported as secreted mammalian chaperone functioning in an ATP-independent manner^24^. In addition to functioning as an extracellular chaperone, Clu has been proposed to play many roles in the cytosol^25^, including regulating tumorigenesis^26^ and mitochondrial-mediated apoptosis^27,28^, and its accumulation has been associated with neuronal degeneration, Alzheimer’s disease, and aging^29^. Furthermore, Clu may act as a suppressor or enhancer of pathology^30^. Although *Clu* is markedly upregulated in oHSCs^31,32^, it is unclear whether Clu can actively regulate the function of oHSCs. To validate our screen results, sgRNAs targeting *Clu* (sgClu) and cDNAs encoding *Clu* open reading frame were cloned into lentiviral vectors for KO or overexpression (OE), respectively. *Cd38* KO and OE were performed in parallel for comparison. To test the effects of *Clu* on HSC differentiation in vitro, cultured Cas9-expressing oHSCs and wild-type (WT) yHSCs were respectively infected with lentiviruses to knock out or overexpress *Clu*. Eight days after infection, cells were sorted and quantified based on the expression of myeloid markers (FcR^+^/Mac-1^+^) or megakaryocyte ratios (Extended Data Fig. 2a). *Clu* KO oHSCs exhibited decreased myeloid differentiation with no effect on megakaryocyte differentiation ratios. Conversely, *Clu* OE yHSCs showed increased myeloid differentiation and increased megakaryocyte ratios (Extended Data Fig. 2b,c). Similar results were obtained for *Cd38* KO and OE (Extended Data Fig. 2b, c). Interestingly, *Clu* OE exhibited a more significant effect on myeloid differentiation compared with that of *Cd38*. Furthermore, colony-forming unit (CFU) assay confirmed the decreased myeloid differentiation potential with *Clu* KO oHSCs exhibiting decreased unipotent mature colonies (BFU-E and CFU-GM) and increased multipotent immature colonies (CFU-GEMM) compared with the control (Extended Data Fig. 2d), indicating loss of myeloid differentiation and enhanced quiescence in *Clu* KO oHSCs. Collectively, these results demonstrate that both Clu and Cd38 have roles in promoting myeloid and megakaryocyte differentiation in vitro.

**Fig. 2:**
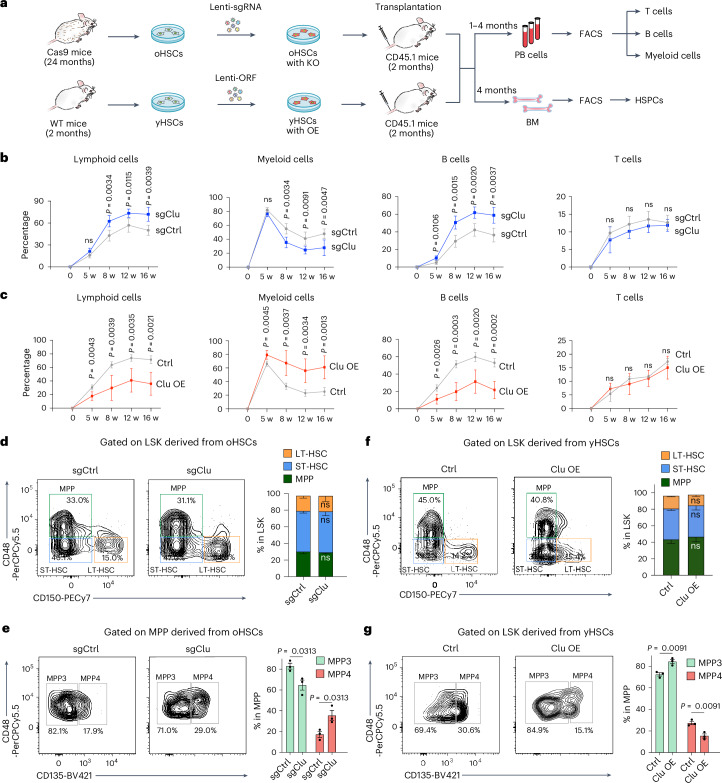
Manipulation of Clu levels in HSCs affects their differentiation. **a**, Workflow for testing the role of Clu in regulating HSC lineage commitment in vivo. **b**,**c**, Frequency of lymphoid, myeloid, B, and T cells in mCherry^+^ peripheral blood cells at different time points after transplantation in recipient mice with *Clu* KO (**b**) or *Clu* OE (**c**) (*n* = 5 per group). **d**, FACS profile (left) and relative abundance of the HSPC populations (right) in the mCherry^+^ LSK (Lin^−^ Sca1^+^ cKit^+^) derived from *Clu* KO oHSCs 4 months after transplantation in recipient mice compared with control mice (*n* = 3 per group). HSPC populations include LT-HSC (Cd150^+^ Cd48^−^), ST-HSC (Cd150^−^ Cd48^−^), and MPP (Cd150^−^ Cd48^+^). **e**, FACS profile (left) and relative abundance of the subpopulations (right) in the mCherry^+^ MPPs derived from *Clu* KO oHSCs 4 months after transplantation in recipient mice compared with control mice (*n* = 3 per group). MPP subpopulations include MPP3 (Cd135^−^ Cd48^+^) and MPP4 (Cd135^+^ Cd48^+^). **f**, FACS profile (left) and relative abundance of the HSPC populations (right) in the mCherry^+^ LSK (Lin^−^ Sca1^+^ cKit^+^) derived from *Clu* OE yHSCs 4 months after transplantation in recipient mice compared with control mice (*n* = 3 per group). **g**, FACS profile (left) and relative abundance of the subpopulations in the mCherry^+^ MPPs derived from *Clu* OE yHSCs 4 months after transplantation in recipient mice compared with control mice (*n* = 3 per group). Unpaired two-tailed Student’s *t*-test (**b**–**g**). Error bars represent mean ± standard deviation (s.d.) (**b,c**) or mean ± standard error of the mean (s.e.m.) (**d**–**g**). HSPC, hematopoietic stem and progenitor cell; ns, not significant; w, weeks. Source data

**Extended Data Fig. 3:**
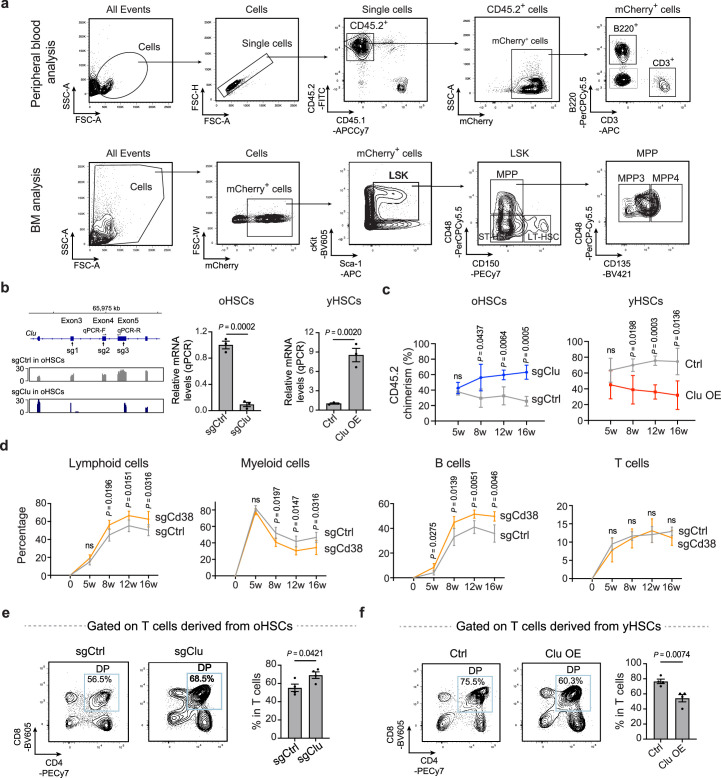
Manipulation of *Clu and Cd38* levels in HSCs affect differentiation of oHSCs *in vivo.* (**a**) FACS profile and cell populations in the peripheral blood derived from HSCs infected with lentivirus after 4 months of transplantation in the recipient mice (top panel). FACS profile and cell populations of HSPCs derived from HSCs infected with lentivirus after 4 months of transplantation in the recipient mice (bottom panel). (**b**) Validation of *Clu* KO (3 sgRNA simultaneously targeting Exon3-5) in oHSCs by RNA-seq (left panel). Validation of *Clu* KO in oHSCs (middle panel) and *Clu* OE in yHSCs (right panel) by qPCR. (**c**) Leukocyte chimerism in the peripheral blood over time after competitive transplantation in the recipient mice with KO or OE of *Clu* in HSCs (n = 4 per group). (**d**) Frequency of lymphoid, myeloid, B, and T cells in mCherry^+^ peripheral blood cells at different time points post-transplantation in recipient mice with *Cd38* KO oHSC (n = 5 per group). (**e**) Representative FACS plot (left) and quantification (right) of CD4^+^/CD8^+^ DP T cells in the thymus of recipient mice with control (sgCtrl) or *Clu* KO (sgClu) oHSCs (n = 4 per group). (**f**) Representative FACS plot (left) and quantification (right) of CD4^+^/CD8^+^ DP T cells in the thymus of recipient mice with control or *Clu* OE yHSCs (n = 4 per group). Unpaired two-tailed Student’s t-test (**b**–**f**). Error bars represent mean ± SEM (**b**, **e**, **f**). Error bars represent mean ± SD (**c**, **d**). Ns: no significant difference. Source data

We next evaluated Clu’s role in vivo. *Clu* KO or OE HSCs were transplanted into recipient mice, and peripheral blood and bone marrow compositions were analyzed (Fig. 2a and Extended Data Fig. 3a). *Clu* KO resulted in increased lymphoid, decreased myeloid, and increased B cell output of oHSCs, indicating that Clu positively contributes to myeloid-biased differentiation (Fig. 2b and Extended Data Fig. 3b). Consistently, OE of *Clu* in yHSCs resulted in a decreased lymphoid, increased myeloid, and decreased B cell output (Fig. 2c and Extended Data Fig. 3b). These results demonstrate that manipulating *Clu* levels in HSCs influences relative lymphoid and myeloid outputs. Because loss of long-term reconstitution capacity is a feature of aged HSCs, we evaluated the effect of *Clu* KO in oHSCs and *Clu* OE in yHSCs on their reconstitution capacity. *Clu* KO in oHSCs resulted in increased peripheral blood chimerism over time, suggesting improved engraftment capacity and long-term reconstitution potential. In contrast, *Clu* OE in yHSCs resulted in decreased chimerism, indicating a deficiency in engraftment capacity (Extended Data Fig. 3c). Similar but weaker effects were obtained with *Cd38* manipulation (Extended Data Fig. 3d).

In addition to myeloid-biased differentiation, age-associated thymic involution resulting in immune senescence also contributes to immune dysfunction^33^. A reduction in the CD4^+^/CD8^+^ double-positive (DP) T cell population in the thymus of aged mice indicates severe thymic involution^34^. Thus, we analyzed changes in thymic subpopulations of recipient mice transplanted with *Clu* KO oHSCs or *Clu* OE yHSCs. We found *Clu* KO in oHSCs resulted in an expansion of DP T cells, whereas *Clu* OE in yHSCs resulted in a decline in DP T cells compared with controls (Extended Data Fig. 3e,f), indicating improved T cell output and hematopoiesis in terms of T cell development in the thymus with *Clu* KO, as well as the converse with *Clu* OE.

Collectively, these results demonstrate that upregulation of *Clu* in oHSCs contributes to sustained myeloid-biased differentiation, and loss of function of *Clu* shifts differentiation toward the lymphoid lineage.

### Manipulation of Clu in HSCs affects progenitor frequencies

In addition to peripheral blood, we analyzed the effect of *Clu* manipulation in HSCs on multipotent progenitors (MPPs) cell differentiation. To this end, hematopoietic stem and progenitor cells (HSPCs) were isolated from the bone marrow of recipient mice 4 months after transplantation (Fig. 2a). Using a described gating strategy^35,36^, long-term HSCs (LT-HSCs), short-term HSCs (ST-HSCs), and MPPs were analyzed by FACS. MPP subpopulations were further separated into CD135^−^ myeloid-primed progenitors (MPP3) and CD135^+^ lymphoid-primed progenitors (MPP4). *Clu* KO in oHSCs and *Clu* OE in yHSCs did not alter the relative percentage of LT-HSC, ST-HSC, or MPP (Fig. 2d,f). However, *Clu* KO in oHSCs decreased the myeloid-primed MPP3 and increased the lymphoid-primed MPP4 percentage compared with control (Fig. 2e), whereas *Clu* OE in yHSCs increased MPP3s and decreased MPP4s (Fig. 2g), indicating that *Clu* expression induces changes in the ratio of the myeloid-primed and lymphoid-primed MPPs.

Collectively, these results demonstrate that Clu plays an important role in regulating HSC differentiation, and a deficiency of Clu increases differentiation of oHSCs into lymphoid-primed progenitors. Conversely, OE of *Clu* increases the differentiation of yHSCs into the myeloid-primed progenitors.

### Clu affects mitochondria morphology and metabolism

**Fig. 3:**
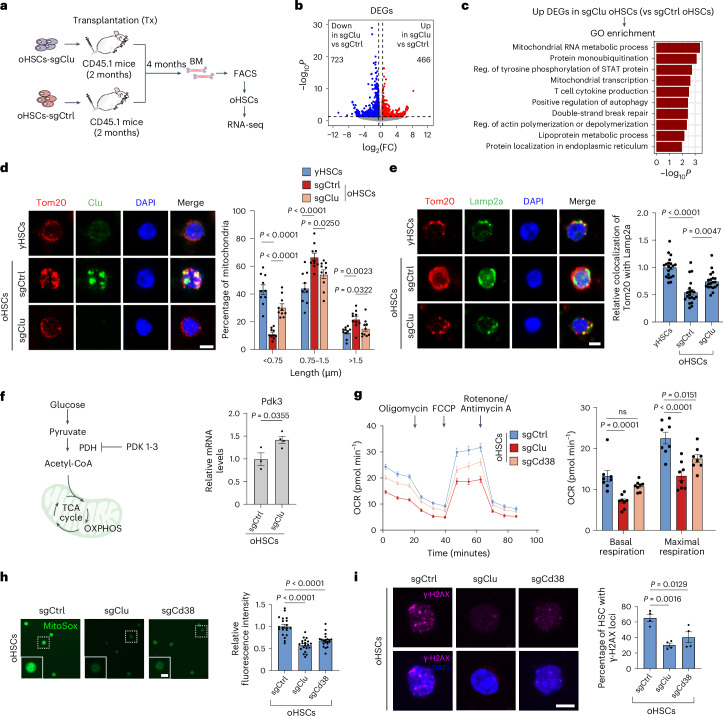
Clu regulates mitochondria function in aged HSCs. **a,** Experimental scheme for analyzing *Clu* KO effects on oHSCs’s transcriptome 4 months after transplantation. **b**, Volcano plot showing DEGs after *Clu* KO in oHSCs. Two-sided Wald test *P* value. **c**, GO enrichment analysis of the terms associated with upregulated genes in *Clu* KO oHSCs. Reg., regulation. **d**, Clu and Tom20 immunofluorescence images (left) and quantification of mitochondrial length in HSCs (*n* = 10 imaging fields containing 20 cells per group) (right). Scale bar, 5 μm. **e**, Lamp2a and Tom20 immunofluorescence images (left) and quantification of co-localization in HSCs (*n* = 20) (right). Scale bar, 5 μm. **f**, Scheme of the glucose to TCA cycle and the relative mRNA levels of *Pdk3* in response to *Clu* KO in oHSCs determined by RT-qPCR (sgCtrl: *n* = 3; sgClu: *n* = 4). **g**, Oxygen consumption rates (OCR) in oHSCs of *Clu* KO, *Cd38* KO, and Ctrl were measured under basal conditions, followed by subsequent addition of the oligomycin, FCCP, and rotenone/antimycin A, as indicated (left). Basal respiration and maximal respiration levels were calculated (right) (*n* = 8 per group). **h**, Labeling (left) and quantification (right) of ROS levels with MitoSox (*n* = 20 per group). Scale bar, 5 μm. **i,** Nuclear γ-H2AX immunofluorescence images (left) and quantification of percentage of HSCs with γ-H2AX loci (right) (*n* = 4 per group). Scale bar, 5 μm. One-way ANOVA with Tukey’s multiple comparison test (**d**,**e**). Unpaired two-tailed Student’s *t*-test (**f**). One-way ANOVA with Dunnett’s multiple comparison test (**g**–**i**). Error bars represent mean ± s.e.m. (**d**–**i**). Source data

**Extended Data Fig. 4:**
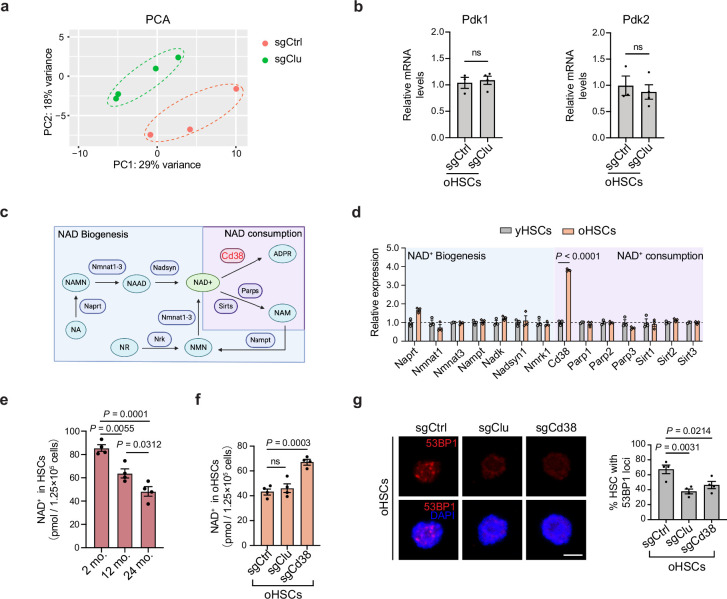
Clu and Cd38 knockout regulate mitochondria function in aged HSCs. (**a**) Principal component analysis (PCA) (left) of the RNA-seq of control and *Clu* KO oHSCs isolated from the recipient mice after 4 months of transplantation (sgCtrl: n = 3; sgClu: n = 4). (**b**) Relative mRNA levels of *Pdk1*, *Pdk2* in control and *Clu* KO oHSCs determined by RT-qPCR (sgCtrl: n = 3; sgClu: n = 4). (**c**) Diagram showing the enzymes involved in NAD^+^ biogenesis and consumption. (**d**) RT-qPCR data showing the mRNA levels of the enzymes involved in NAD^+^ biogenesis and consumption in yHSCs and oHSCs (n = 3 per group). (**e**) NAD^+^ levels in HSCs from 2, 12, 24 months-old mice (n = 4 per group). (**f**) NAD^+^ levels in control, *Clu* or *Cd38* KO oHSCs (n = 4 pergroup). (**g**) Nuclear 53BP1 immunofluorescence images (left) and quantification of the percentage of oHSCs with 53BP1 loci (right) (n = 4 per group). Scale bar, 5 μm. Unpaired two-tailed Student’s t-test (**b**, **d**). One-way ANOVA with Tukey’s multiple comparison test (**e**). One-way ANOVA with Dunnett’s multiple comparison test (**f**, **g**). Error bars represent mean ± SEM (**b**, **d**, **e**–**g**). Ns: no significant difference. Source data

To understand how *Clu* KO in oHSCs leads to reversal of myeloid-biased differentiation, we performed RNA sequencing (RNA-seq) of oHSCs isolated from the bone marrow of recipient mice 4 months after transplantation (Fig. 3a and Extended Data Fig. 4a) and identified 723 down- and 466 upregulated genes (Fig. 3b and Supplementary Table 2). Gene ontology (GO) enrichment analysis of the upregulated genes identified multiple terms related to mitochondrial functions, including mitochondrial RNA metabolic process, mitochondrial transcription and positive regulation of autophagy (Fig. 3c), suggesting that Clu may regulate mitochondrial function in oHSCs.

Clu accumulation has been previously associated with aging and aging-related diseases^37^. However, the role of Clu in HSCs and more specifically in aged HSCs is unknown. Given its potential role in mitochondria, we examined the location of Clu protein in oHSCs. We found that Clu is mostly localized with mitochondria, as it largely overlaps with translocase of the outer mitochondrial membrane 20 (Tom20), a marker for the morphology of mitochondrial networks^38^ (Fig. 3d). Using a published method^39^, we quantified mitochondria length in HSCs. Interestingly, oHSCs have more mitochondria with a longer average length and a more complex network compared with yHSCs, suggesting a hyperfused mitochondria network in oHSCs. Importantly, Clu depletion in oHSCs reduced the average mitochondria length (Fig. 3d, right panel).

Functional mitochondrial fission machinery allows for proper segregation of dysfunctional components of mitochondrial networks and facilitates their degradation via mitophagy^12,40^. Based on the autophagy-related GO term (Fig. 3c) and the finding that increased Clu causes mitochondria fusion (Fig. 3d), we hypothesized that accumulation of hyperfused mitochondrial networks in oHSCs is accompanied by a decline in mitophagy. To test this hypothesis, we measured co-localization of Tom20 with the lysosomal marker Lamp2a, an indicator of mitophagy flux^41^. *Clu* KO both reduced the mitochondria network (Tom20 staining) and increased the co-localization of Tom20 with Lamp2a in oHSCs (Fig. 3e), indicating that loss of Clu reduces hyperfused mitochondria and facilitates mitophagy for the clearance of damaged mitochondria.

Mitochondria play an essential role in maintaining metabolic homoeostasis. Both stress and aging can disrupt metabolic homoeostasis, resulting in increased oxidative phosphorylation (OXPHOS) and metabolic activity, which was concomitant with impaired stem cell function^42^. In addition, pyruvate dehydrogenase kinases 3 (Pdk3) play key roles in metabolic checkpoints for HSC quiescence and stem cell maintenance^22^. Pyruvate dehydrogenase kinases can negatively regulate the glucose to OXPHOS switch by inhibiting pyruvate dehydrogenase (PDH) complex activity to prevent pyruvate from entering TCA cycling^22^ (Fig. 3f, left). Interestingly, *Clu* KO in oHSCs increased *Pdk3* mRNA levels but did not affect *Pdk1* or *Pdk2* (Fig. 3f and Extended Data Fig. 4b), which prompted us to analyze whether *Clu* KO results in a metabolic change. Using the Seahorse mito stress assay, we found that *Clu* KO resulted in a lower basal respiration and maximal respiration in oHSCs (Fig. 3g). This reduced OXPHOS metabolic rate implies a switch of aged HSCs back to the quiescent metabolic state, a characteristic of young LT-HSCs^15^.

Nicotinamide adenine dinucleotide (NAD^+^) is a central mediator of metabolic processes that plays an essential role in maintaining metabolic homoeostasis^43^. Analysis of the enzymes involved in NAD^+^ biogenesis and consumption indicated that only *Cd38* expression is significantly increased in oHSCs compared with yHSCs (Extended Data Fig. 4c,d). Consistent with age-associated increase of Cd38, the NAD^+^ level exhibited an age-associated decline in HSCs (Extended Data Fig. 4e). The decrease in NAD^+^ level is due to the age-associated Cd38 increase in HSCs, as KO of *Cd38*, but not *Clu*, resulted in a significant increase in the NAD^+^ level (Extended Data Fig. 4f). These results indicate that Cd38 depletion contributes to mitochondrial homeostasis through increasing NAD^+^ level. Consistent with its role in mitochondria homeostasis^15^, *Cd38* KO also reduced the maximal respiration in oHSCs with a less pronounced effect compared with *Clu* KO (Fig. 3g).

The increased OXPHOS in oHSCs with *Clu* and *Cd38* upregulation prompted us to measure ROS, as they are key features of aging and the mitochondrial metabolic pathway^44^. We found that *Clu* or *Cd38* KO reduced ROS production as indicated by decreased MitoSox labeling (Fig. 3h). Because ROS can induce DNA damage^45^, we measured DNA damage by staining the double stranded break marker γ-H2AX and found that *Clu* or *Cd38* KO reduced DNA double stranded breaks (Fig. 3i). Consistently, *Clu* or *Cd38* KO also decreased the signal of 53BP1 (Extended Data Fig. 4g), a DNA double stranded break binding protein^46^. Collectively, these data indicate that *Clu* or *Cd38* KO reduces OXPHOS, ROS, and DNA damages.

In summary, these results demonstrate that Clu plays a crucial role in regulating mitochondrial function during HSC aging. *Clu* KO switches the metabolism of oHSCs towards that of yHSCs with reduced OXPHOS, which improves the fitness of mitochondrial metabolism.

### Clu promotes mitochondrial fusion by interacting with Mfn2

**Extended Data Fig. 5:**
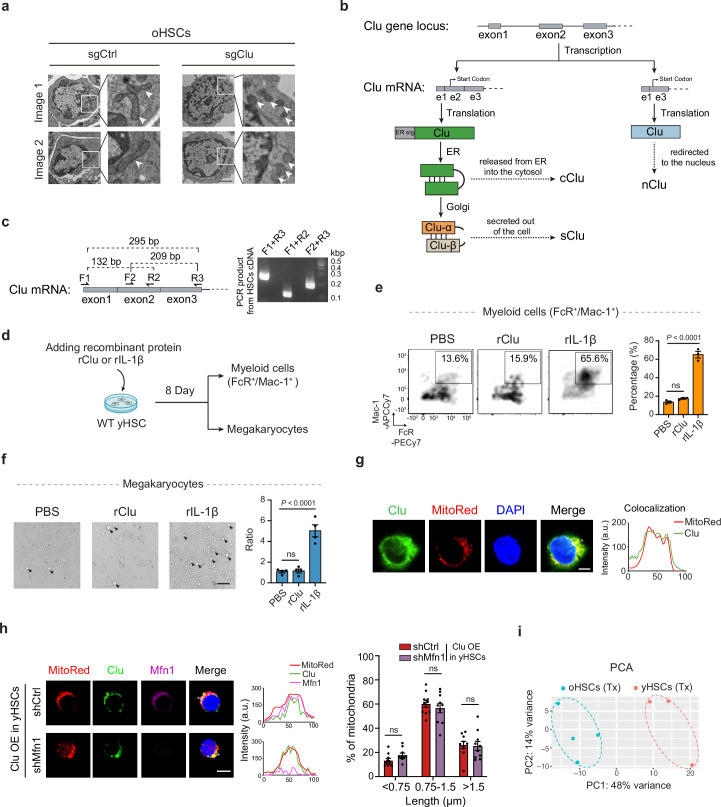
Extracellular Clu is not responsible for promoting myeloid output. (**a**) Transmission electron microscopy images of *Clu* KO and control oHSCs collected from the recipient mice after 4 months of transplantation. Scale bar, 2 μm. Magnified views of the mitochondria are shown on the right indicated by white arrowheads. (n = 10 imaging fields containing 20 cells per group). (**b**) Schematic representation of Clu isoforms. (**c**) Schematic of the murine Clu locus from exon1 to exon3. Electrophoresis analysis of the PCR products using cDNA templates derived from mRNA of HSCs. Sig, signal peptide. (**d**) Workflow for verification of the role of extracellular Clu in regulating HSC differentiation in vitro. Recombinant Clu (rClu) and IL-1βwere separately added to the culture medium of yHSCs. Cells were collected after 8 days of culture for flow cytometry analysis using myeloid markers (FcR^+^/Mac-1^+^) and imaging of megakaryocytes. (**e**) Representative FACS plots (left) and quantification (right) of FcR^+^/Mac-1^+^ cells were shown for PBS, rClu (25 ng/ml) or rIL-1β (25 ng/ml) treated yHSCs after differentiation culture (n = 3 per group). (**f**) Representative images (left) and quantification (right) of megakaryocyte ratio for PBS, rClu (25 ng/ml) or rIL-1β (25 ng/ml) treated yHSCs after differentiation culture (n = 4/group). Scale bar, 100 μm. (**g**) Immunofluorescence images (left) and fluorescence intensity profile plots (right) of HSCs overexpressing cClu-v5, co-stained with MitoRed (n = 10 imaging fields containing 20 cells per group). Scale bar, 2 μm. (**h**) Immunofluorescence images of mitochondria (MitoRed), cClu-v5, and Mfn1 in shCtrl and shMfn1 yHSCs overexpressing cClu (left). Fluorescence intensity profile plots (middle) in arbitrary units (a.u.) along the solid lines, and quantification (right) of mitochondria length by MitoRed (n = 10 imaging fields containing 20 cells per group). Scale bar, 5 μm. (**i**) Principal component analysis (PCA) of the RNA-seq of young and old HSCs isolated from the recipient mice 4 months after transplantation (yHSCs: n = 3; oHSCs: n = 4). One-way ANOVA with Dunnett’s multiple comparison test (**e**, **f**). Unpaired two-tailed Student’s t-test (**h**). Error bars represent mean ± SEM (**e**, **f**, **h**). Ns: no significant difference. Source data

To understand how *Clu* KO affects mitochondrial function, we performed transmission electron microscopy analysis and found that oHSCs with *Clu* KO had small and round mitochondria, whereas the Ctrl oHSC had elongated and swollen mitochondria (Extended Data Fig. 5a), which is consistent with our immunofluorescence data showing a reduced average mitochondria length with *Clu* KO (Fig. 3d). This indicates that Clu may play a role in regulating mitochondrial morphological dynamics.

Although Clu was reported as an extracellular chaperone, subsequent studies have demonstrated its roles in the cytosol^25,47^. After translation, Clu is transported to the endoplasmic reticulum (ER) lumen, where it undergoes the removal of the 22-mer ER signal peptide. After transportation to the Golgi, Clu is cleaved into alpha and beta subunits linked through disulfide bonds. This heterodimeric form (secreted Clu (sClu)) is then secreted out of the cells. However, under stress, non-cleaved full-length Clu is directly released from the ER into the cytosol (cytoplasmic Clu (cClu)) to perform its intracellular functions (Extended Data Fig. 5b)^25,47^. Interestingly, Clu can tightly attach to mitochondria to inhibit apoptosis by interacting with activated Bax in cancer cells^48^. Clu also disrupts mitochondrial distribution patterns in COS-7 cells^28^. Furthermore, Clu translated without exon2 has been reported to localize in the nucleus (nClu) to regulate cellular apoptosis^47,49^ (Extended Data Fig. 5b). Thus, it is important to determine the Clu isoforms present in oHSCs to understand how it performs it function in the context of aging.

PCR using cDNAs from HSCs followed by electrophoresis revealed the presence of exon2, indicating that the nClu form is not present in HSCs (Extended Data Fig. 5c). Because *Clu* OE increased the myeloid output of yHSCs (Extended Data Fig. 2b,c), we asked whether extracellular sClu has the same effect by adding recombinant murine Clu (rClu) to the culture medium of WT yHSCs followed by analyzing myeloid output after 8 days of culture (Extended Data Fig. 5d). Compared with the PBS control, the presence of rClu did not alter the Mac-1^+^/FcR^+^ myeloid output, whereas treatment with IL-1β, a known myeloid differentiation inducer^50^, significantly increased the myeloid output (Extended Data Fig. 5e). Similar results were also obtained in megakaryocytes (Extended Data Fig. 5f). These results indicate that intracellular Clu, but not extracellular Clu, is responsible for the increased myeloid output observed with *Clu* OE in yHSCs.

**Fig. 4:**
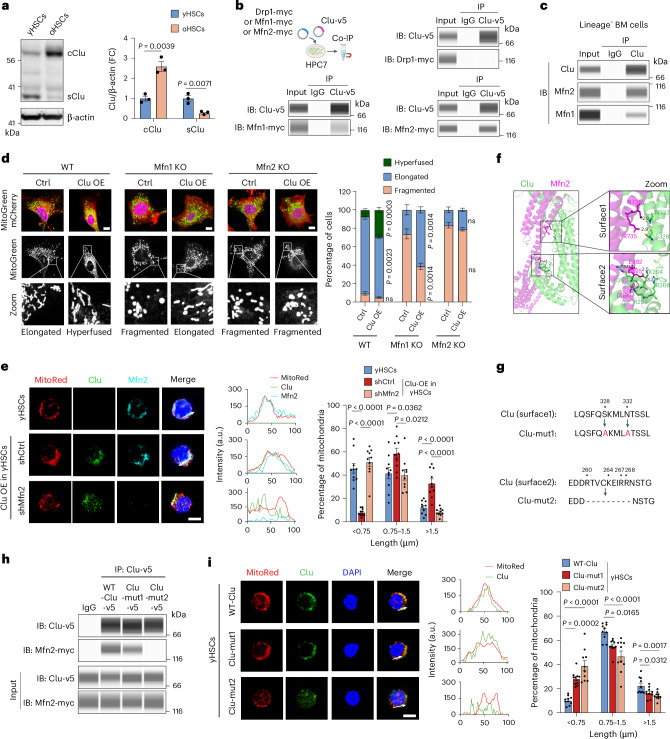
Clu specifically interacts with Mfn2 to enhance mitochondrial fusion. **a**, Immunoblot (left) and quantification (right) of cytoplasmic Clu (cClu) and secreted Clu (sClu) in yHSC and oHSC lysates (*n* = 3 per group). **b**, Co-immunoprecipitation (co-IP) analyses of cClu-v5 with Drp1-10 × myc, Mfn1-10 × myc or Mfn2-10 × myc overexpressed in HPC7 cells (*n* = 3). Immunoblots (IBs) were performed using automated ProteinSimple WES capillary electrophoresis system. **c**, Co-IP analysis of endogenous Clu with Mfn1 and Mfn2 in lineage negative (lineage^−^) bone marrow cells (*n* = 3). Immunoblots were performed using the ProteinSimple WES. **d**, Immunofluorescence images (left) and quantification (right) of mitochondria (MitoGreen) in WT, *Mfn1* KO and *Mfn2* KO MEFs without (Ctrl) or with cClu overexpression (*Clu* OE). MEFs were transduced with mCherry control or cClu-T2A-mCherry lentivirus (*n* = 4 per group). T2A was used to separate Clu and mCherry into two proteins. Scale bar, 10 μm. **e**, Immunofluorescence images of mitochondria (MitoRed), cClu-v5, and Mfn2 in shCtrl and shMfn2 yHSCs overexpressing cClu (left). Fluorescence intensity profile plots in arbitrary units (a.u. along the solid lines (middle) and quantification (right) of mitochondria length by MitoRed (*n* = 10 imaging fields containing 20 cells per group). Scale bar, 5 μm. **f**, Cytoplasmic Clu (UniProt: Q06890) and Mfn2 (UniProt: Q80U63) interacting surfaces predicted by AlphaFold 3. **g**, The amino acid sequences and their corresponding missense and deletion mutations (mut1 and mut2) on Clu surfaces predicted to be involved in Mfn2 interaction. **h**, Co-IP analyses of WT-cClu-v5, cClu-mut1-v5, or cClu-mut2-v5 with Mfn2-10 × myc. Clu mutants were separately overexpressed with Mfn2 in HPC7 cells (*n* = 3). Immunoblots were performed using the ProteinSimple WES. **i**, Immunofluorescence images of mitochondria (MitoRed) in WT-cClu, cClu-mut1, and cClu-mut2 overexpressed yHSCs (left). Fluorescence intensity profile plots in arbitrary units (a.u.) along the solid lines (middle) and quantification (right) of mitochondria length by MitoRed (*n* = 10 imaging fields containing 20 cells per group). Scale bar, 5 μm. Unpaired two-tailed Student’s *t*-test (**a**,**d**). One-way ANOVA with Tukey’s multiple comparison test (**e**,**i**). Error bars represent mean ± s.e.m. (**a**,**d**,**e**,**i**). Source data

Given that Clu is retained in the cytosol under ER stress^51^, we postulated that aging-associated stress might cause retention of Clu in the cytosol. Western blot analysis indicated that the non-cleaved full-length cClu is increased whereas the cleaved sClu is decreased in oHSCs compared with yHSCs (Fig. 4a), indicating a shift from sClu to cClu with aging. The concurrent increase in cClu and mitochondrial hyperfusion in oHSCs (Fig. 3d) prompted us to ask whether cClu localizes to mitochondria. To this end, we infected HSCs with lentivirus expressing the full-length cClu with a v5-tag, which lacks the ER signal peptide to prevent secretion. Immunostaining confirmed that the cClu-v5 co-localized with mitochondria in HSCs (Extended Data Fig. 5g), consistent with mitochondria localization of endogenous Clu in aged HSCs.

Because Clu regulates mitochondrial morphological dynamics, we asked whether Clu interacts with regulators of mitochondrial fusion or fission. To this end, we co-expressed a v5-tagged cClu with a myc-tagged mitochondria fusion factor, Mfn1 or Mfn2, or a fission factor, Drp1, in the HSPC cell line HPC7 (Fig. 4b). Co-immunoprecipitation followed by western blot analysis demonstrated that Clu-v5 strongly interacted with Mfn2-myc and weakly interacted with Mfn1-myc but did not interact with Drp1-myc (Fig. 4b). We next asked whether the endogenous Clu interacts with Mfn2 or Mfn1 in bone marrow lineage negative HSPCs. Immunoprecipitation using a Clu antibody not only immunoprecipitated Clu, but also Mfn2 (strongly) and Mfn1 (weakly) (Fig. 4c), suggesting that Clu could promote mitochondria fusion by interacting with Mfn2 and Mfn1.

Next, we asked whether Clu-stimulated mitochondrial fusion requires the presence of Mfn1 and Mfn2. We tested this in mouse embryonic fibroblasts (MEF) cells, as MEFs allow for monitoring of mitochondrial morphology in a Mfn1 and Mfn2 dependent manner^39,52^. We assessed mitochondrial morphology (hyperfused, elongated or fragmented) in the presence or absence of *Clu* OE using *Mfn1* KO or *Mfn2* KO MEF cell lines (Fig. 4d). *Clu* OE resulted in hyperfused mitochondria with increased length compared with WT control (Fig. 4d, left imaging panel). Increased fragmentation was observed in *Mfn1* or *Mfn2* KO MEFs (Fig. 4d, middle and right imaging panels). In *Mfn1* KO MEFs, *Clu* OE significantly increased the percentage of elongated mitochondria (Fig. 4d, middle imaging panel), but *Clu* OE failed to achieve this effect in *Mfn2* KO MEFs (Fig. 4d, right imaging panel), suggesting that Clu-stimulated mitochondrial fusion requires Mfn2.

We next asked whether the same is true in HSCs. We constructed lentivirus simultaneously overexpressing *Clu* with shRNAs silencing *Mfn1* or *Mfn2*. Clu localization and mitochondrial morphology were then analyzed. Compared with control yHSCs, *Clu* OE showed a concomitant decrease in short mitochondria (<0.75 μm) and an increase of 0.75 to 1.5 μm and >1.5 μm mitochondria (Fig. 4e). Importantly, shRNA-mediated *Mfn2* knockdown decreased the co-localization of Clu with mitochondria and impaired Clu’s ability to promote mitochondrial fusion (Fig. 4e). However, *Mfn1* knockdown did not cause a change in the localization of Clu or mitochondrial length (Extended Data Fig. 5h), indicating that Mfn2 is required for Clu to induce mitochondrial hyperfusion in HSCs.

To further confirm the role of Clu-Mfn2 interaction in mitochondrial hyperfusion, we used AlphaFold 3 (ref. ^53^) to predict the interaction between Mfn2 and Clu. We identified two interaction surfaces (surface 1 and surface 2) on Clu (Fig. 4f) and generated two Clu mutants with mutations at these surfaces (Fig. 4g). Co-immunoprecipitation analysis following exogenous expression of tagged proteins demonstrated that the surface 1 missense mutation (Clu-mut1) reduced Clu’s interaction with Mfn2, whereas the surface 2 deletion mutation (Clu-mut2) completely abolished the Clu-Mfn2 interaction (Fig. 4h). Importantly, the surface 2 Clu mutant that fails to interact with Mfn2 also lost its ability to co-localize with mitochondria and fails to increase the mitochondrial length when compared with WT Clu (Fig. 4i), confirming that Clu-Mfn2 interaction is required for Clu’s role in stimulating mitochondrial hyperfusion.

Collectively, these results demonstrate that Clu plays a crucial role in regulating mitochondrial morphological changes during HSC aging through interaction with the fusion factor Mfn2.

### Loss of Clu downregulates p38 MAPK signaling in oHSCs

**Fig. 5:**
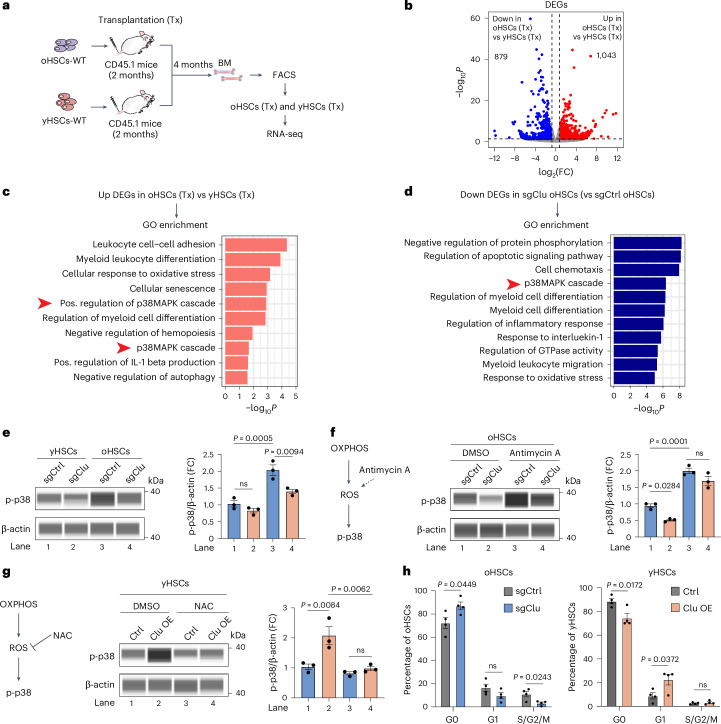
Clu activates the p38 MAPK pathway in oHSCs. **a**, Experimental scheme for comparing transcriptomes of oHSCs and yHSCs 4 months after transplantation (Tx). **b**, Volcano plot showing DEGs between transplanted oHSCs and yHSCs. Two-sided Wald test *P* value. **c**, Enriched GO terms of upregulated genes in transplanted oHSCs compared with yHSCs. **d**, Enriched GO terms of downregulated genes in *Clu* KO (sgClu) oHSCs compared with control oHSCs (sgCtrl). Pos., positive. **e**, Immunoblot (left) and quantification (right) of p-p38 protein levels in yHSCs and oHSCs with or without *Clu* KO (*n* = 3 per group). Immunoblots were performed using the ProteinSimple WES. **f**, Immunoblot (left) and quantification (right) of p-p38 protein levels in control and *Clu* KO oHSCs with or without antimycin A treatment (*n* = 3 per group). Immunoblots were performed using the ProteinSimple WES. **g**, Immunoblot (left) and quantification (right) of p-p38 protein levels in control and *Clu* OE yHSCs with or without *N*-acetylcysteine (NAC) treatment (*n* = 3 per group). Immunoblots were performed using the ProteinSimple WES. **h**, Cell cycle distribution of control (sgCtrl) or *Clu* KO (sgClu) oHSCs (left). Cell cycle distribution of control (Ctrl) or *Clu* OE yHSCs (right) (*n* = 4 per group). One-way ANOVA with Tukey’s multiple comparison test (**e**–**g**). Unpaired two-tailed Student’s *t*-test (**h**). Error bars represent mean ± s.e.m. (**e**–**h**). Source data

Mitochondrial fusion counteracts metabolic insults by enhancing OXPHOS and preventing mitophagy^54^. Consistently, we found that *Clu* KO in oHSCs not only increased mitophagy (Fig. 3e) but also decreased OXPHOS and ROS (Fig. 3g, h). In addition to causing DNA damage, ROS regulates cell signaling in different cell types^55^. To further understand how *Clu* expression might regulate HSC differentiation, we performed RNA-seq analysis comparing oHSCs with yHSCs after transplantation and identified 1,043 upregulated genes (Fig. 5a,b, Extended Data Fig. 5i and Supplementary Table 3). Interestingly, in addition to the enrichment of myeloid differentiation and response to oxidative stress, GO analysis of the 1,043 upregulated genes in oHSCs revealed the enrichment of the p38 MAPK pathway (Fig. 5c), suggesting that this pathway might be activated during aging. Further analysis of the downregulated genes in response to *Clu* KO in oHSCs showed the enrichment of the p38 MAPK pathway (Fig. 5d), indicating that *Clu* upregulation in oHSCs might be responsible for p38 activation. These observations combined with previous findings that the p38 MAPK pathway is activated by ROS, DNA damage, and cytokine release^56^, as well as the observation that increased ROS can abrogate the reconstituting capacity of HSCs^57^, suggest that Clu might regulate HSC differentiation by activating p38. To investigate whether p38 is activated by increased Clu in oHSCs, we examined the active form of p38 (phosphorylated p38 (p-p38)) levels and found that the p-p38 levels were higher in oHSCs compared with that in yHSCs (Fig. 5e, lane 3 vs lane 1). Importantly, *Clu* KO downregulates the p-p38 level in oHSCs (Fig. 5e, lane 4 vs lane 3). Increasing ROS through antimycin A treatment further elevated p-p38 levels (Fig. 5f, lane 3 vs lane 1), indicating that p-p38 can sense changes in ROS. Importantly, antimycin A-induced ROS increase compensated the decrease of p-p38 caused by *Clu* KO (Fig. 5f, lane 4 vs lane 2), indicating ROS is downstream of Clu. Conversely, inhibiting ROS by treatment with the antioxidant *N*-acetylcysteine (NAC) blocked Clu OE induced p-p38 increase in yHSCs (Fig. 5g, lane 4 vs lane 2).

The p38 MAPK pathway regulates cell cycle transitions, thus affecting HSC quiescence^58^. During transplantation, quiescent donor HSCs are induced to proliferate and differentiate in recipient bone marrow, and most will return to quiescence to avoid exhaustion^59^. However, inducing HSCs to a continuously proliferative state leads to HSC exhaustion^2^. To assess the effect of Clu manipulation on cell state, we analyzed the cell cycle phase distribution of *Clu* KO oHSCs and *Clu* OE yHSCs 16 weeks after transplantation. *Clu* KO in oHSCs resulted in increased percentages of cells in G0 phase, whereas *Clu* OE in yHSCs resulted in a decrease in the G0 phase compared with controls (Fig. 5h). These results indicate that *Clu* KO facilitates the maintenance of oHSCs in a quiescent state, whereas *Clu* OE in yHSCs promotes their exit from a quiescent state (G0) to enter the cell cycle.

Collectively, *Clu* KO changed the metabolism of oHSC towards that in yHSC with reduced OXPHOS and downregulated p-p38 levels by reducing intracellular ROS. Thus, our data support the existence of a Clu-OXPHOS-p38 signaling pathway in aged HSCs.

### Clu regulates myeloid-biased hematopoiesis through Cebpb

**Fig. 6:**
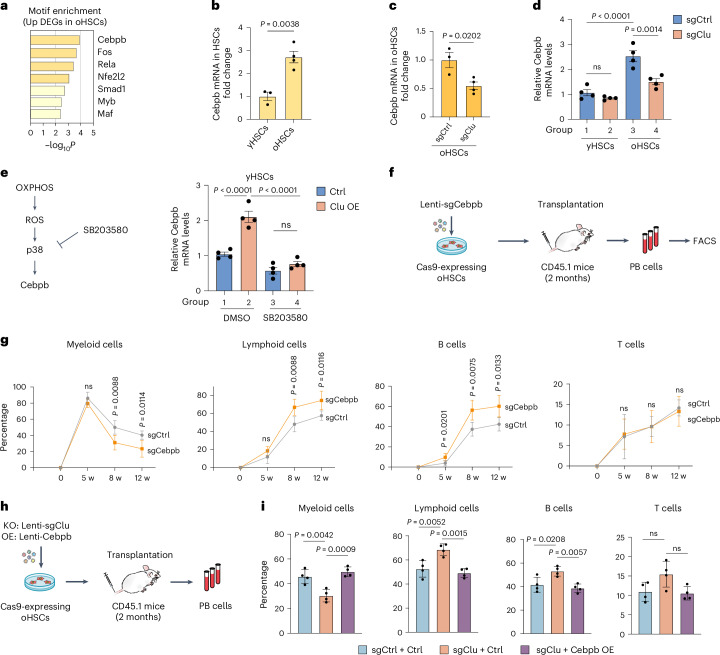
Clu regulates myeloid-biased hematopoiesis through p38 and Cebpb. **a**, Heatmap of transcription factor motif enrichment based on the upregulated genes in oHSCs compared with yHSCs. **b**, Relative *Cebpb* mRNA levels in yHSCs and oHSCs measured by RNA-seq (yHSCs: *n* = 3; oHSCs: *n* = 4). **c**, Relative *Cebpb* mRNA levels in control and *Clu* KO oHSCs measured by RNA-seq (sgCtrl: *n* = 3; sgClu: *n* = 4). **d**, RT-qPCR analysis of *Cebpb* mRNA levels in yHSCs and oHSCs with or without *Clu* KO (*n* = 4 per group). **e,** RT-qPCR analysis of *Cebpb* mRNA levels in control and *Clu* OE yHSCs with or without SB203580 treatment (*n* = 4 per group). **f**, Schematic of HSC transplantation after *Cebpb* KO in oHSCs. **g**, Frequency of myeloid, lymphoid, B, and T cells in mCherry^+^ peripheral blood cells at different time points after transplantation with *Cebpb* KO oHSCs in recipient mice (*n* = 5 per group). **h**, Schematic of HSC transplantation after *Clu* KO with or without *Cebpb* OE in oHSCs. **i**, Frequency of myeloid, lymphoid, B, and T cells in mCherry^+^ peripheral blood cells at 3 months after transplantation with *Clu* KO combined with or without *Cebpb* OE oHSCs in recipient mice (*n* = 4 per group). Unpaired two-tailed Student’s *t*-test (**b,c,g**). One-way ANOVA with Tukey’s multiple comparison test (**d,e,i**). Error bars represent mean ± s.e.m. (**b**–**e**); mean ± s.d. (**g**,**i**). Source data

Consistent with myeloid-biased differentiation of oHSCs, analysis of the upregulated genes in oHSCs revealed enrichment of myeloid-related terms (Fig. 5c). However, *Clu* KO reduced the genes related to myeloid cell differentiation in oHSCs (Fig. 5d). To determine how *Clu* upregulation in oHSCs contributes to myeloid differentiation, we focused on transcriptional factors (TFs), which are known to play critical roles in regulating lineage commitment of HSCs^60^. We applied Metascape to predict the candidate TFs that regulate the upregulated genes in oHSCs, which revealed Cebpb as a top candidate TF (Fig. 6a). Consistently, Cebpb-deficient mice exhibited reduced myelocytes^61^, suggesting that Cebpb may play an important role in myeloid cell differentiation during aging. RNA-seq also revealed that *Cebpb* is upregulated in oHSCs compared with that in yHSCs (Fig. 6b). Importantly, *Clu* KO decreased the *Cebpb* mRNA level (Fig. 6c). Real-time quantitative PCR (RT-qPCR) further confirmed the downregulation of *Cebpb* mRNA after *Clu* KO in oHSCs (Fig. 6d). Collectively, these data suggest that Clu might regulate myeloid bias through upregulation of *Cebpb*.

Because p38 activation promotes the expression or activation of transcription factors^62^, we next asked whether *Cebpb* is upregulated by *Clu* OE, and if so, whether it is mediated by p38 activation. We found that *Cebpb* is upregulated by *Clu* OE (Fig. 6e, lane 2 vs lane 1), and this effect is blocked by the presence of a p38 inhibitor (SB203580) (Fig. 6e, lane 4 vs lane 2), indicating that Clu-mediated p38 activation is responsible for Cebpb upregulation.

Next, we asked whether myeloid bias in aged HSCs could be alleviated by decreasing Cebpb expression in vivo. To this end, we infected Cas9-expressing oHSCs with lentiviruses expressing sgRNA that target *Cebpb* and transplanted the *Cebpb* KO oHSCs into recipient mice (Fig. 6f). Peripheral blood analysis revealed that *Cebpb* KO in oHSCs is sufficient to decrease myeloid differentiation and increase B cell differentiation (Fig. 6g). To further demonstrate that Cebpb is a downstream factor mediating the effect of Clu, we asked whether reduced myeloid differentiation by *Clu* KO in oHSCs can be restored by overexpressing *Cebpb* (Fig. 6h). HSC transplantation followed by peripheral blood analysis demonstrated that the decreased myeloid or increased lymphoid differentiation in *Clu* KO was completely reversed by ectopic expression of *Cebpb* (Fig. 6i), highlighting the role of Cebpb in myeloid-biased differentiation triggered by *Clu* upregulation in oHSCs.

Taken together, these results provide strong evidence that upregulation of Clu in oHSCs regulates differentiation by activating p38, which in turn upregulates Cebpb causing myeloid-biased differentiation.

### Clu loss of function rejuvenates aged hematopoietic system

**Fig. 7:**
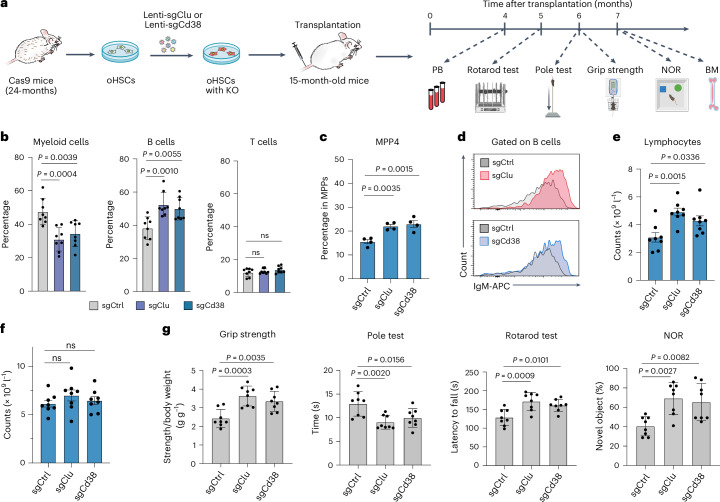
Clu loss of function rejuvenates aged HSCs and systemic mouse functionality. **a**, Strategy for testing the in vivo effect of *Clu* or *Cd38* KO in oHSCs transplanted into 15-month-old mice. Timeline of the blood and behavioral tests after transplantation of oHSCs with *Clu* or *Cd38* KO. Behavioral test icons in a created with BioRender.com. **b**, Frequency of myeloid, B, and T cells in mCherry^+^ peripheral blood cells at 4 months after transplantation with *Clu* or *Cd38* KO oHSC in the recipient mice (*n* = 8 per group). **c**, Frequency of MPP4 cells in bone marrow at 7 months after transplantation with *Clu* or *Cd38* KO oHSCs in the recipient mice (*n* = 4 per group). **d**, Histogram of IgM^+^ B cells at 4 months after transplantation with *Clu* or *Cd38* KO oHSCs in the recipient mice. **e**,**f**, Total lymphocyte and white blood cell numbers after transplantation with *Clu* or *Cd38* KO oHSCs in the recipient mice (*n* = 8 per group). **g**, Behavioral tests of recipient mice transplanted with *Clu* and *Cd38* KO oHSCs. Grip strength: measurements of the ratio of the mouse strength to body weight. Pole test: measurements of the total time the mice used to climb down from top to bottom. Rotarod test: measurements of the latency to fall. Novel object recognition (NOR): measurements of the rate of exploring a novel object (*n* = 8 per group). One-way ANOVA with Dunnett’s multiple comparison test (**b**,**c**,**e**,**f**,**g**). Error bars represent ± s.d. (**b**,**g**) or mean ± s.e.m. (**c**,**e**,**f**). Source data

Having revealed the mechanism of Clu in myeloid-biased differentiation, we next asked whether we can correct the myeloid bias to achieve rejuvenation by depleting Clu. To this end, we isolated Cas9-expressing HSCs from aged mice followed by transduction with lentiviruses expressing sgRNAs targeting *Clu* and then transplanted them into 15-month-old recipients. After 4 to 7 months, peripheral blood, immune cells, and physical functions of the recipient mice were analyzed to evaluate the effects of *Clu* KO (Fig. 7a). Because *Cd38* KO has similar effects on oHSC differentiation (Extended Data Fig. 3d), we also performed parallel experiments with *Cd38* KO. We found *Clu* or *Cd38* KO resulted in decreased myeloid and increased B cell outputs (Fig. 7b), as well as increased MPP4 and IgM producing B cells (Fig. 7c,d). Importantly, both *Clu* and *Cd38* KO increased the absolute number of lymphocytes without changing the total white blood cell counts (Fig. 7e,f). The improved hematopoiesis and immune system also resulted in better physical (grip strength test, pole test, rotarod test) and brain (novel object recognition test) functions (Fig. 7g), indicating loss function of *Clu* or *Cd38* in oHSCs has a rejuvenation effect while *Cd38* KO exhibiting a weaker effect than *Clu* KO (Fig. 7b,g).

**Extended Data Fig. 6:**
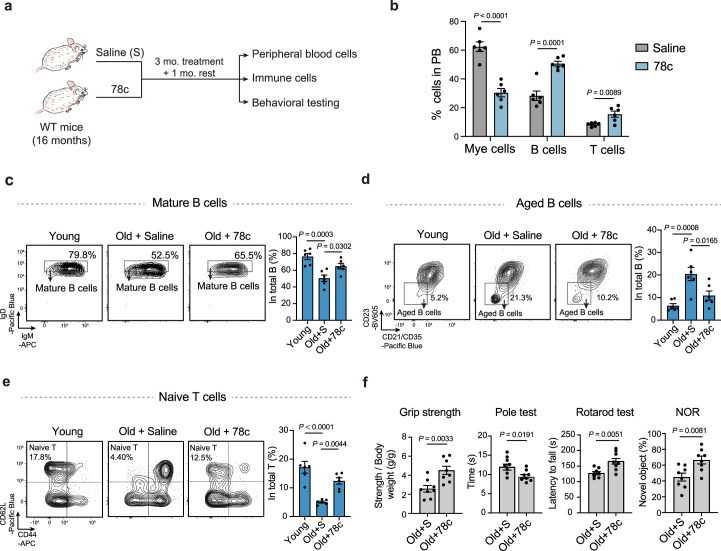
Effects of inhibition of *Cd38* on hematopoiesis. (**a**) Scheme for testing the treatment of mice with saline (S) or 78c. (**b**) Frequency of myeloid, B, and T cells in mice at 4 months after the treatment with saline or 78c (n = 6 per group). (**c**) FACS plots (left) and quantification (right) of mature B cells (IgM^+^ IgD^+^) as the percentage of total B cells in the blood of mice treated with saline or 78c (n = 6 per group). (**d**) FACS plots (left) and quantification (right) of aged B cells (CD21/CD35^−^, CD23^−^) as the percentage of total mature B cells in the blood of mice treated with saline or 78c (n = 6 per group). (**e**) FACS plots (left) and quantification (right) of naive T cells (CD44^−^ CD62L^+^) as the percentage of total CD4^+^ and CD8^+^ T cells in the blood of mice treated with saline or 78c (n = 6 per group). (**f**) Physical performance assessed by grip strength, pole test, rotarod test and novel object recognition in mice treated with saline or 78c (n = 8 per group). Unpaired two-tailed Student’s t-test (**b**, **f**); One-way ANOVA with Tukey’s multiple comparison test (**c**–**e**). Error bars represent mean ± SEM (**b**–**f**). Ns: no significant difference. Source data

To evaluate the translational potential of our findings, we asked whether administration of an available potent Cd38 chemical inhibitor 78c^63^ is able to correct the myeloid bias and improve immune and physical function. To this end, 78c was administered to 16-month-old mice daily for 3 months. After a 1-month rest, peripheral blood composition, functional immune cells, and physical functions were analyzed (Extended Data Fig. 6a). We found that 78c treatment significantly decreased myeloid cell and increased lymphoid cell output (Extended Data Fig. 6b). Aged immune systems undergo accumulation of exhausted cells and reduced generation of T and B lymphocytes with immune decline^64^. To evaluate the effect of 78c in restoring youthful immune features, we examined the T and B cell subsets and found that mature B cell frequency is increased in old mice with 78c treatment compared with the saline control, whereas the frequency of aged B cells is decreased (Extended Data Fig. 6c,d). Similarly, the frequency of naive T cells is increased after 78c treatment (Extended Data Fig. 6e). These results indicate that 78c treatment corrected the age-related myeloid bias and improved the immune system. Importantly, 78c treatment also improved physical and brain functions (Extended Data Fig. 6f), highlighting 78c as a potential rejuvenation agent.

## Discussion

Aging is associated with increased myelopoiesis and decreased lymphopoiesis, resulting in an impaired immune system that is defective in responding to pathogens^65^. To understand the mechanisms underlying these aging-related hematopoietic defects, we performed a CRISPR-based screen and identified Clu, whose upregulation in oHSCs drives myeloid-biased differentiation. We demonstrate that Clu promotes mitochondrial hyperfusion through interaction with Mfn2, which promotes hyperactive mitochondrial OXPHOS with increased ROS production. This in turn activates p38 MAPK, leading to *Cebpb* upregulation to drive myeloid-biased differentiation. We further demonstrate that KO of *Clu* in oHSCs alleviates myeloid-biased differentiation and improves immune function, leading to whole-body rejuvenation. Our study identifies Clu as a driver of aging-related defects and reveals an OXPHOS-p38-Cebpb axis through which Clu mediates myeloid-biased differentiation of oHSCs, suggesting a possible targeting strategy for rejuvenation. Further work will be required to comprehensively understand the impact of Clu on OXPHOS.

Although Clu has been reported to be upregulated in oHSCs^31,32^, it was unclear whether it actively regulates the function of oHSCs. Here we provide several lines of evidence suggesting that Clu is a driver of myeloid-biased differentiation in oHSCs. First, *Clu* is expressed at a low level in yHSCs and is upregulated in oHSCs (Fig. 1c). Second, *Clu* KO reduced myeloid differentiation and increased lymphoid differentiation in oHSCs (Fig. 2b), whereas *Clu* OE in yHSCs increased myeloid differentiation and reduced lymphoid differentiation (Fig. 2c). Third, *Clu* KO decreased the percentage of myeloid-primed MPP3 and increased the percentage of lymphoid-primed MPP4 (Fig. 2e). Conversely, *Clu* overexpression resulted in the opposite effects (Fig. 2g). Differences in the relative percentage of myeloid- or lymphoid-primed MPPs in response to changes in *Clu* expression likely explain the biased myeloid and lymphoid outputs in peripheral blood. Our results are consistent with a recent study demonstrating that Clu-positive HSCs progressively expand with aging and display an increased propensity for myeloid-biased differentiation^66^. Manipulation of *Cd38* also resulted in similar results (Extended Data Fig. 3d), consistent with a recent report^67^.

Analysis of the upregulated genes from *Clu* KO revealed a link to mitochondria function. Although Cd38 has been linked to mitochondria through NAD^+^ metabolism, how Clu regulates mitochondrial function in HSCs was unknown. Interestingly, hyperfused mitochondria are greatly reduced in response to *Clu* KO (Fig. 3d), indicating that Clu serves as a mitochondrial stress regulator in aged HSCs. Importantly, *Clu* KO increased the localization of mitophagy marker Lamp2a to mitochondria in oHSCs (Fig. 3e), indicating *Clu* KO improves mitochondria fitness by increasing mitophagy for the damaged mitochondria clearance. Strikingly, *Clu* KO in oHSCs significantly reduced the basal and maximal respiration (Fig. 3g), as well as ROS and DNA damage (Fig. 3h,i). These changes in mitochondria functions support the notion that *Clu* KO might revert oHSCs back to a more quiescent state with lower respiration and metabolism, which are characteristics of yHSCs^42^.

Clu has different isoforms with varied localization in cells^25,47^. However, cClu is the major form in oHSCs (Fig. 4a). Several lines of evidence support that upregulation of Clu in oHSCs promotes mitochondria hyperfusion through Mfn2 interaction. First, cClu co-localizes with mitochondria in HSCs. Second, Clu physically associates with the mitochondrial fusion proteins Mfn2 and Mfn1 (Fig. 4b,c). Third, Clu’s co-localization with mitochondria and Clu-stimulated mitochondria fusion are both dependent on Mfn2 (Fig. 4e). Finally, disruption of the Clu–Mfn2 interaction abolished Clu’s capacity to stimulate mitochondrial fusion (Fig. 4i), demonstrating a key role of Mfn2 in mediating Clu’s function. Consistent with previous proposals that Clu functions as an adaptor in promoting hetero-protein complex stability^68,69^, our finding that Clu promotes mitochondrial hyperfusion through interacting with Mfn2 to form a fusion complex provides further evidence, supporting that Clu functions by promoting hetero-protein complex stability.

Given that increased ROS abrogates the reconstituting capacity of HSCs through ROS-induced activation of p38 MAPK^57^, we asked whether Clu-mediated OXPHOS and ROS elevation can activate p38 and found that the p-p38 levels were higher in oHSCs compared with yHSCs, and *Clu* KO reduced the p-p38 level in oHSCs (Fig. 5e). We also provided several lines of evidence demonstrating that upregulation of *Cebpb* by p-p38 is responsible for myeloid-biased differentiation in oHSCs. First, Cebpb binding motif is the most enriched in the promoters of upregulated genes in oHSCs compared with yHSCs (Fig. 6a), and *Cebpb* is upregulated in oHSCs compared with yHSCs (Fig. 6b). Second, *Cebpb* is downregulated in response to *Clu* KO (Fig. 6c,d). Third, Clu-mediated *Cebpb* upregulation can be blocked by p38 inhibition (Fig. 6e). Fourth, *Cebpb* KO in oHSCs reduced myeloid and increased lymphoid output (Fig. 6g), and finally, *Clu* KO-induced differentiation changes are blocked by *Cebpb* OE (Fig. 6i). Collectively, our study reveals how Clu mediates mitochondrial hyperfusion and promotes myeloid-biased differentiation in oHSCs via an OXPHOS-p38-Cebpb axis.

We also assessed the long-term effect of *Clu* KO in oHSCs by transplantation, which revealed that *Clu* KO in oHSCs not only reduced the aging-associated myeloid bias and increased the lymphoid population but also improved physical and brain functions (Fig. 7b–g). Similarly, *Cd38* KO in oHSCs or treatment with a Cd38 inhibitor 78c resulted in balanced differentiation, improved immune proportion and improved physical and brain function (Fig. 7b–g and Extended Data Fig. 6). Our results are in line with 78c as a rejuvenation agent^63^ and are consistent with recent studies demonstrating that Cd38 inhibition improves aging-related ovary functional decline, maintains mouse fertility^70^ and extends mice lifespan^71^. However, our study additionally demonstrates that 78c alleviates aging-associated myeloid bias and increases functional immune cells. Importantly, our results indicate that *Clu* KO has a more pronounced effect compared with *Cd38* KO (Fig. 7b–g), highlighting Clu as a potential therapeutic target for rejuvenation.

**Extended Data Fig. 7:**
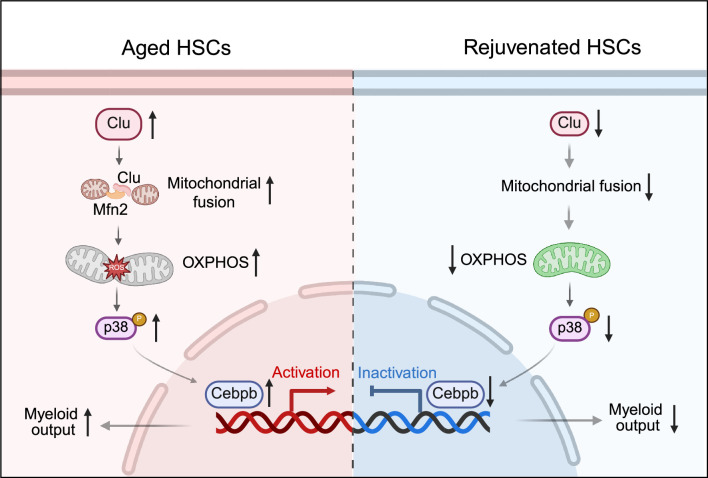
Schematic illustration of the mechanism by which loss of Clu function attenuates myeloid-biased differentiation in aged HSCs. Age-dependent increase in Clu expression leads to an increase in mitochondrial fusion through interaction with Mfn2. This in turn facilitates excessive mitochondrial OXPHOS with ROS, which activates the p38 MAPK signaling pathway resulting in increased Cebpb expression. As a key transcription factor driving myeloid cell differentiation, upregulation of Cebpb leads to myeloid-biased differentiation in aged HSCs. Depletion of Clu can reduce the biased differentiation and alleviate age-associated functional decline through reducing mitochondrial hyperfusion, improving mitophagy and restoring youthful metabolism. Illustration created with BioRender.com.

Collectively, our study identified Clu as a regulator of mitochondrial fusion and revealed the OXPHOS-p38-Cebpb axis through which Clu regulates myeloid differentiation bias in oHSCs (Extended Data Fig. 7). *Clu* KO attenuates aged HSCs and whole-body functional decline. Although our study is performed in mice, aging-induced Clu upregulation has been reported in human HSCs^72^ and hematopoietic progenitor cells^73^. Given that aging-induced Clu upregulation is conserved in human HSCs, targeting Clu can be a potential therapeutic intervention in aging-related functional decline.

## Methods

### Mouse strains and cell line

All experiments with animals were conducted in accordance with the National Institutes of Health's *Guide for the Care and Use of Laboratory Animals* and approved by the IACUC of the Boston Children’s Hospital and Harvard Medical School. 24-month-old male and female Rosa26-Cas9 knock-in mice were used for the CRISPR–Cas9-based screen and individual gene KO. Two-month-old male and female Rosa26-Cas9 knock-in mice (# 026179), as well as the 2-month-old and >18-month-old C57BL/6J male and female mice (# 000664), were purchased from the Jackson Laboratory. Two-month-old CD45.1 male and female mice (# 002014) were purchased from the Jackson Laboratory. All mice were kept under specific pathogen-free conditions within an environment controlled for temperature (20–22 °C) and humidity (40–70%) and were subjected to a 12-h light/dark cycle.

HEK293T cells (ATCC, CRL-3216) were grown in Dulbecco’s modified Eagle medium (DMEM, Thermo Fisher, cat #11-960-069) with 10% fetal bovine serum (FBS, Corning, cat #35010CV). HPC7 lines were routinely maintained in IMDM (Thermo Fisher Scientific, #12440053) supplemented with 10% FBS, 1.5 × 10^−4^ M MTG and SCF.

### Plasmid cloning and lentiviral packaging

The pCF221 with five barcodes encoding the U6-sgRNA and mCherry was digested with *BsmB*I (NEB, R0580) and gel extracted (Zymo Research, D4008). Three sgRNAs targeting different exons of each candidate gene were designed by Benchling (www.benchling.com). All 90 sgRNA oligos (Supplementary Table 4) with adapter sequences were synthesized by IDT and pooled in equal molarity. The pooled sgRNA oligos were amplified and cloned into the *BsmB*I-digested vector. Ligated vectors were transformed into competent cells (NBE, C3040H) and cultured for 10 h. Plasmids were extracted using the ZymoPURE II Plasmid Purification Kit (Zymo Research, D4202), and the sgRNAs in the plasmids were verified by sequencing.

Three sgRNAs targeting each gene in our CRISPR screen were chosen for individual gene KO. The sgRNAs were ordered from IDT and cloned individually into the *Bsm*BI-digested vector. Each cloned vector was verified by Sanger sequencing using the human U6 promoter sequencing primer. For each gene, three plasmids containing the sgRNAs were co-transfected into 293T cells. Lentivirus packing were performed in HEK293T cells and purified by Lenti-X concentrator (Takara, # 631232) or ultracentrifugation.

For Clu, Cebpb OE in HSCs, Clu ORF or Cebpb ORF gBlock was synthesized by IDT and then cloned into pCF221 with mCherry. For co-immunoprecipitation, cClu (without the 22-mer signal) was synthesized by IDT. cClu was fused with a v5 tag at the C-terminus and cloned into pCF221 without mCherry. Mfn1-10 × Myc (#23212), Mfn2-10 × Myc (#23213), and pcDNA3.1(+)-Drp1 (#34706) were purchased from Addgene. Mfn1-10 × Myc and Mfn2-10 × Myc were amplified by PCR and cloned into pCF221 individually. Drp1 was cloned into pCF221 and another 10 × myc was added to the C-terminus of Drp1. cClu-mut1 has two missense mutations on surface 1, and cClu-mut2 has a deletion on surface 2. cClu-mut1 and cClu-mut2 were synthesized by IDT and cloned into pCF221 without mCherry separately. All cloning was performed using DNA Assembly Master Mix (NEB, #E2621S).

For simultaneous OE of cClu and knockdown of Mfn1 or Mfn2 in HSCs, shRNA sequences targeting Mfn1 or Mfn2 were cloned downstream of the U6 promoter in pCF221-cClu-v5 vector by replacing the sgRNA cassette. The high scored shRNA sequence was designed by the Genetic Perturbation Platform (GPP) of Broad Institute.

To overexpress cClu in MEFs, cClu was fused to mCherry at the C-terminus and T2A was inserted between Clu and mCherry to allow for the cleavage of both proteins. The cClu-T2A-mCherry fragment was cloned into pCF221.

### HSC isolation, sorting and culture

The humerus, pelvic, femur, and tibia bones of the mice were isolated. The bones were cut into fine pieces and suspended in 1 × PBS (Gibco, 10010-023). The solution was filtered with a 70 µm cell strainer. The cells were centrifuged at 400 *g* for 10 min and resuspended with PBS in a FACS tube. HSPCs were isolated via magnetic separation using Streptavidin RapidSpheres from the EasySep Mouse Hematopoietic Progenitor Cell Isolation Kit (StemCell Technologies, 19856) according to the manufacturer’s instructions. The isolated cells were centrifuged at 400 g and resuspended with PBS. After staining, the cells were sorted with SH800S Cell Sorter (Sony Biotechnology) and cultured in plates containing DMEM F12, 1 × P/S/G, 10 mM HEPES, 1 mg ml^−1^ PVA, 1 × ITSX, 100 ng ml^−1^ TPO, 10 ng ml^−1^ SCF. The cells were incubated at 37 °C in 5% CO_2_. All antibodies used are listed in Supplementary Table 5.

### CFU assay

Bone marrow cells were isolated from recipients transplanted with *Clu* KO oHSCs after 16 weeks, and 3 × 10^5^ cells were plated in methylcellulose medium (MethoCult GF M3434, StemCell Technologies) and incubated at 37 °C in 5% CO_2_ for 12 days. Colonies were imaged using the Keyence BZ-X800 microscope (Keyence) and scored by morphology according to the manufacturer’s manual.

### Pooled CRISPR screening and transplantation

FACS sorted HSCs from aged Cas9 mice were infected with mCherry^+^ lentivirus for 12 h. Two days after infection, mCherry^+^ HSCs were FACS sorted on a SH800S Cell Sorter (Sony Biotechnology). 150,000 HSCs were collected through FACS for each replicate in the screen. The remaining cells after FACS sorting were collected for genomic DNA extraction, amplification of sgRNA with barcode regions, and deep-sequencing analysis as the sgRNA distribution in start-point oHSCs.

In preparation for transplantation^74^, recipient mice were lethally irradiated at a rate of 1,000 rad. Twelve hours after irradiation, donor cells were transplanted into recipient mice along with 2 × 10^5^ helper cells via lateral tail vein injections. The recipient mice were given water containing 1 mg ml^−1^ neomycin (RPI) for 2 weeks.

For the repopulation assays, mCherry^+^ HSCs were sorted following infection with sgRNA or OE lentivirus. CD45.2 *Clu* KO oHSCs or *Clu* OE yHSCs were transplanted with CD45.1 competitor cells into lethally irradiated CD45.1 recipient mice. Cd45.2 *Cd38* KO oHSCs or *Cd38* OE yHSCs were transplanted in separate experiments using the same method. Overall donor chimerism and donor-derived lymphoid and myeloid ratios in peripheral blood were analyzed over a 16-week period.

### Peripheral blood and bone marrow cell analysis

At 4 weeks’ time intervals, 30 µl of peripheral blood was collected from the mice in tubes containing 3 µl of 0.5 M EDTA (Thermo Fisher, cat #R1021). 300 µl of 1 × Red Blood Cell Lysis Buffer (Invitrogen, cat #00-4333-57) was added and incubated at room temperature (RT) for 10 min. The cells were washed and resuspended with 1 × PBS. After antibody staining (Supplementary Table 5), peripheral blood was analyzed using SH800S Cell Sorter (Sony Biotechnology) or LSR Fortessa (BD Biosciences).

At the end of the transplantation experiment, bone marrow cells were extracted as described above. The gating strategy used for discriminating different cell types (LT-HSCs, ST-HSCs, MPPs) was adapted from the published methods^35,36^.

### Thymocyte analysis

Thymus was dissociated through a 70 µm cell strainer. The cells were centrifuged at 400 *g* for 8 min, resuspended, and stained with anti-CD3, CD4, CD8 and CD45.2 antibodies (Supplementary Table 5). The samples were incubated on ice for 30 min before FACS analysis using BD Fortessa.

### Cell cycle analysis

Young WT and old Cas9-expressing HSCs were harvested from donor mice and transduced via lentivirus to express either *Clu* OE or sgClu, respectively. The mCherry^+^ cells were transplanted into lethally irradiated CD45.1 young (2 months) recipient mice. After 4 months, bone marrow cells were collected from the recipient mice, and HSPCs were isolated using the EasySep Mouse Hematopoietic Progenitor Cell Isolation Kit (StemCell Technologies, 19856). After staining with HSC marker antibodies, cells were fixed and stained with Ki67 and Hoechst33342 according to the manufacturer’s instruction for Cyto-Fast Fix/Perm Buffer Set (BioLegend, #426803) and analyzed using FACS (LSR Fortessa).

### Library preparation for screening sequencing

Five months after transplantation, all peripheral blood was collected from the recipient mice into tubes containing EDTA (Thermo Fisher, cat #R1021). Cells were lysed with 1× Red Blood Cell Lysis Buffer (Invitrogen, cat #00-4333-57), washed and stained with CD11b-Pacific blue, CD45.2-FITC, CD3-APC, B220-PerCP-Cy5.5 and CD45.1-APC-Cy7 for 30 min at 4 °C. FACS sorting was performed using SH800S Cell Sorter (Sony Biotechnology). HSCs were also collected and FACS sorted as endpoint oHSCs. Genomic DNA was extracted from the sorted cells using the QIAamp DNA Micro Kit (Qiagen, #56304). sgRNA sequencing library construction was performed using a two-step protocol. Guide RNAs with the barcode coding region in gDNA were amplified by Q5 High-Fidelity DNA Polymerase (NEB, M0491S) and purified. Then the PCR product was used as input for a second step introducing the standard Illumina adapters. The final Illumina libraries were pooled and subjected to high-throughput sequencing. The read count of each sgRNA was calculated with no mismatches and aligned to the sequence of reference sgRNA. Data was analyzed using MAGeCK^75^ with default RRA parameters.

### Mitochondrial metabolism analysis

Seahorse Mito Stress Test was performed according to the manufacturer’s manual (XFe96 Analyzer, Agilent Technologies). The day prior to the assay, 10,000 HSCs were seeded onto 0.01% poly-ʟ-lysine (PLL)-coated 96-well plates with HSC growth medium and incubated at 37 °C overnight. On the day of the assay, the growth medium was replaced with the Seahorse DMEM assay medium (pH 7.4, 0.5× P/S, 2 mM L-glutamine, 1 mM pyruvate, 3 mg ml^−1^ glucose, 100 ng ml^–1^ TPO, 10 ng ml^−1^ SCF). Cells were incubated for 1 h at 37 °C without CO_2_. The drugs were prepared and loaded onto the cartridge (2 µM oligomycin, 1.5 µM FCCP, 0.5 µM rotenone/antimycin A). After calibration, oxygen consumption rate was measured with the XFe96 Analyzer under the following injection strategy: 3 min mix, 0 min wait, 4 min measure and 3 × repeats.

### NAD^+^ analysis

NAD^+^ level was analyzed using the NAD/NADH colorimetric assay kit from Abcam (ab65348). HSCs were lysed with NAD extraction buffer with two freeze/thaw cycles. The lysate was centrifuged at top speed for 5 min at 4 °C. The supernatant was collected and filtered with a 10 kD spin column (ab93349). NAD^+^ was decomposed by heating the samples at 60 °C for 30 min followed by immediate cooling on ice. Reaction mix was prepared by mixing the NAD cycling buffer and NAD cycling enzyme mix from the kit. Reaction mix, samples, developer solution were added onto a 96-well plate in order. The plate was incubated at RT, and NAD^+^ levels were measured using the CLARIOstar Plus microplate reader (BMG Labtech) at 4 h intervals at OD 450 nm.

### Immunofluorescence

Cultured HSCs were infected with lentivirus carrying *Clu* OE and Mfns knockdown expression cassette. HSCs were then seeded onto a 24-well plate with circular cover slips coated with PLL. After 3 days, medium was aspirated from the wells, and the cells were fixed with 4% paraformaldehyde (PFA) and incubated for 10 min. The PFA was aspirated, and the wells were washed with PBS twice. 0.1% Triton X-100 in PBS was added to the wells for 10 min, and TNB buffer was used for blocking for 1 h. Primary antibodies (Supplementary Table 5) were diluted with TNB buffer, added to the wells, and incubated overnight at 4 °C. Cells were washed with PBS and incubated with the fluorescently labeled secondary antibody of the corresponding species. Cells were washed and stained with DAPI. Slides with the cover slips were imaged with confocal microscopy (ZEISS LSM 800; Olympus FV3000R). For other immunofluorescence, cultured HSCs were electroporated with 10 μg recombinant Cas9 Nuclease (IDT, 1081058) with 5 μg synthetic Clu or Cd38 sgRNA (IDT) by Lonza 4D-Nucleofector using primary P3 solution. To measure ROS levels, HSCs were stained with the MitoSOX Green reagent from the Mitochondrial Superoxide Indicator kit (Thermo Fischer Scientific) for 30 min at 37 °C according to the manufacturer’s instructions. Cells were washed with medium and seeded into slides for confocal microscopy imaging (ZEISS LSM 800).

### Image quantification of mitochondrial morphology

WT MEFs (SCRC-1008), Mfn1-null MEFs (CRL-2992) and Mfn2-null MEFs (CRL-2993) were grown in DMEM (Thermo Fisher Scientific, catalogue number 11-960-069) with 10% FBS (Corning, catalogue number 35010CV). For visualization of mitochondria, the MEFs were stained with 400 nM MitoSpy Green FM (BioLegend, number 424805) or MitoSpy Red CMXRos (BioLegend, number 424801) and infected with lentivirus expressing Clu-T2A-mCherry. MEFs were classified based on the morphology of the mitochondrial network^52,76,77^. ‘Elongated’: more than 90% of the mitochondria were tubular; ‘fragmented’: more than 90% of the mitochondria were round; ‘hyperfused’: mitochondrial network was highly reticular and interconnected in MEFs. Data are presented as the percentage of cells in each type from four individual replicate experiments. For mitochondrial length measurements in HSCs, confocal images were collected and projected as a z-project in ImageJ. The Tom20, MitoGreen or MitoRed stained mitochondria were manually traced and classified into bins based on length and presented as percent of total mitochondria^39^. Data presented were from 10 imaging fields (20 cells). For transmission electron microscopy analysis, HSCs collected from the recipient mice were pelleted for 10 min at 4 °C at 600 *g* and fixed in 4 °C overnight in a fix buffer (2% glutaraldehyde, 1% PFA). The remaining steps were completed by the HMS Electron Microscopy Core Facility, and the images were taken using a Tecnai G2 Spirit BioTWIN transmission electron microscope.

### Immunoblotting

Cultured HSCs after gene editing were lysed with RIPA lysis buffer containing protease inhibitor cocktail (Roche, 4693159001) and Halt protease and phosphatase inhibitor cocktail (Thermo Fisher Scientific, 78440). Protein concentrations were measured by CLARIOstar Plus microplate reader (BMG Labtech) using a Pierce BCA Protein Assay Kit (Life Technologies, 23227). Equivalent amounts of samples were loaded and separated by SDS-PAGE (Bolt Bis-Tris Plus Mini Protein Gels, NW04125BOX) and transferred onto PVDF membranes (iBlot 2 Transfer Stacks, IB24002) by iBlot 2 Gel Transfer Device. Then, the membranes were incubated overnight with the primary antibodies (Supplementary Table 5) at 4 °C, followed by incubation with the respective secondary antibody for 1 h at RT. Finally, the immunoreactivity image was detected by iBright CL1500 Imaging System (Thermo Fisher Scientific) using the Immobilon western Chemiluminescent HRP Substrate (Millipore WBKLS0100), and quantification was performed by ImageJ.

As indicated in the figure legend, some immunoblottings were performed using the automated ProteinSimple WES capillary electrophoresis system (Wes; Bio-Techne). In the automatic process, proteins of interest were identified by primary antibodies (Supplementary Table 5), followed by HRP-conjugated secondary antibodies (ProteinSimple). Chemiluminescent signals were detected using the Luminol-Peroxide Mix (ProteinSimple, Biotin Detection Module, #DM-004) and analyzed using the Compass for SW (ProteinSimple) software.

### Co-immunoprecipitation

HPC7 or lineage negative HSPCs were lysed in Pierce IP Lysis Buffer (Thermo Fisher Scientific, #87787) with Halt protease and phosphatase inhibitor cocktail (Thermo Fisher Scientific, #78440). The supernatants were transferred into a new tube after being centrifuged for 20 min at 15,000 *g* at 4 °C. 50 μl supernatants were used as the input. Dynabeads Protein G (Thermo Fisher Scientific, 10004D) were incubated with primary antibody or IgG at 4 °C overnight according to manufacturer’s protocol. The antibody was crosslinked with the magnetic Dynabeads to avoid co-elution before immunoprecipitation. Then, cell lysate was mixed with the bead-antibody complex and rotated at 4 °C overnight. The magnetic bead-protein complex was washed with washing buffer, and proteins were eluted by non-denaturing elution buffer.

### RNA-seq

The procedure from the SMART-Seq v4 Ultra Low Input RNA Kit (Clontech, 634890) was performed according to manufacturer’s instructions. The constructed library was purified and subjected to sequencing (NextSeq 550). The sequencing results were demultiplexed into fastq files. To remove adapters and low-quality reads, fastq data were processed into clean data by TrimGalore (v0.6.7). The clean data were aligned to reference genome sequences (mm10) using Hisat2 (v2.1.0). The gene count matrix was calculated using FeatureCounts (v2.0.1) of the subread package. DEGs were analyzed by DESeq2 package. The *P* value was corrected for multiple testing using the Benjamini and Hochberg method. DEGs between two groups were characterized by *P* value < 0.05 and |log_2_ fold change| > 0.5. Finally, ClusterProfiler package (v4.2.2) was used for identifying enrichment of GO terms.

### Protein structure prediction

Amino acid sequences of murine cClu, which was generated by removing the 22-mer ER signal from the murine Clu, murine Mfn2 and murine Bax were obtained from UniProt and uploaded to AlphaFold 3 for protein-protein interaction prediction. Images of the interaction surfaces were then generated and analyzed using PyMOL.

### RT-qPCR

FACS sorted or cultured HSCs were lysed by TRI Reagent (Zymo, R2050), and the total RNAs were isolated using RNA Clean & Concentrator (Zymo, R1013). cDNA was synthesized with the Superscript III First-Strand Synthesis System (Thermo Fisher Scientific, 18080051) according to the manufacturer’s instructions. RT-qPCR was performed in 96-well plates by QuantStudio 6 Pro Real-Time PCR Systems (Thermo) using Fast SYBR Green Master Mix (Thermo Fisher Scientific, 4385610). The PCR primers are listed in Supplementary Table 6. The Ct values were recorded by QuantStudio 6 Design and analysis software. Relative RNA expression was analyzed by 2^−ΔΔCt^ method and normalized to *Actb* mRNA.

### Behavior tests

#### Grip strength

Forelimb grip strength of the mice was measured using Bioseb’s Grip Strength Test. Mice were first placed onto the hash grid of the instrument, so they are firmly gripped onto the grid. The mice were gently pulled off the grid by their tail and the strength value displayed by the instrument was recorded. The weight of each mouse was also measured and recorded in conjunction with grip strength. All tests were performed on the same day.

#### Pole test

A cylindrical pole (50 cm in height and 1 cm in diameter) used for the pole test was placed vertically upright. Mice were placed facing downwards near the top of the pole. The mice were released, and the time taken to descend to the floor was recorded.

#### Rotarod test

Mice were placed horizontally on the elevated, rotating rod of a rotarod apparatus (Med Associates, ENV-574M) rotating at a constant speed. The length of time that the mice remained on the rod was measured using a timer. The test was repeated three times for each mouse.

#### Novel object recognition test

To test memory, mice underwent three phases as a part of a novel object recognition test. On day 1, the mouse was placed into a closed area and allowed to explore the empty space for 15 min as a part of the habituation phase. On day 2, two identical objects were placed into the enclosure, and the mouse was allowed to explore the objects for 10 min during the training phase. On day 3, one of the previous objects was then replaced with a novel object, and the mouse was allowed to explore both the previous familiar object and the novel object for 10 min during the testing phase. The number of times the mouse contacted each object was recorded.

### Statistics and reproducibility

Statistics was performed using the GraphPad Prism 10 software. The sample sizes were based on previous studies in the field and were not predetermined by any statistical methods. Mice with transplantation failures were excluded from the study. All analyses were performed based on the results from biological and technical replicates. All experiments in this study were repeated three or more times as mentioned in the figure legends. Animals allocated to experimental groups were based on genotype and age. No method of randomization was used to assign mice to experimental groups. Group allocation for initial data collection was not performed blind to the investigators, but the group identities during data analysis were blinded to the investigators. Comparisons between two experimental groups were analyzed using the 2-tailed unpaired Student’s *t*-test. The significance of differences among groups were calculated using one-way ANOVA. The results are expressed as the mean ± s.e.m. or mean ± s.d. A significant difference was defined as a *P* value less than 0.05. The statistical tests used and *P* value for each figure are presented in the figure or figure legend.

### Reporting summary

Further information on research design is available in the Nature Portfolio Reporting Summary linked to this article.

## Supplementary information

Reporting Summary

Supplementary Tables Supplementary Tables1–6.

## Source data

Source Data Fig. 1 Statistical source data.

Source Data Fig. 2 Statistical source data.

Source Data Fig. 3 Statistical source data.

Source Data Fig. 4 Statistical source data unprocessed western blots.

Source Data Fig. 5 Statistical source data unprocessed western blots.

Source Data Fig. 6 Statistical source data.

Source Data Fig. 7 Statistical source data.

Source Data Extended Data Fig. 1 Statistical source data.

Source Data Extended Data Fig. 2 Statistical source data.

Source Data Extended Data Fig. 3 Statistical source data.

Source Data Extended Data Fig. 4 Statistical source data.

Source Data Extended Data Fig. 5 Statistical source data unprocessed gels.

Source Data Extended Data Fig. 6 Statistical source data.

## Data Availability

All the data supporting the findings of the study are available from the corresponding author upon reasonable request. RNA-seq data have been deposited to GEO under the accession number GSE275462. Source data are provided with this paper.
