# Supplementary material for: Clusterin drives myeloid bias in aged hematopoietic stem cells by regulating mitochondrial function
Source: Nat Aging. 2025 Jun 30;5(8):1510–27. doi: 10.1038/s43587-025-00908-z (PMC12350150; doi:10.1038/s43587-025-00908-z)
Supplement: Supplementary file 1 — Reporting Summary [file 43587_2025_908_MOESM1_ESM.pdf]

nature portfolio

Corresponding author(s): Yi Zhang

Last updated by author(s): Apr 26, 2025

Reporting Summary

Nature Portfolio wishes to improve the reproducibility of the work that we publish. This form provides structure for consistency and transparency in reporting. For further information on Nature Portfolio policies, see our Editorial Policies and the Editorial Policy Checklist.

Statistics

For all statistical analyses, confirm that the following items are present in the figure legend, table legend, main text, or Methods section.

n/a	Confirmed	
☐	☒	The exact sample size (*n*) for each experimental group/condition, given as a discrete number and unit of measurement
☐	☒	A statement on whether measurements were taken from distinct samples or whether the same sample was measured repeatedly
☐	☒	The statistical test(s) used AND whether they are one- or two-sided<br>*Only common tests should be described solely by name; describe more complex techniques in the Methods section.*
☒	☐	A description of all covariates tested
☐	☒	A description of any assumptions or corrections, such as tests of normality and adjustment for multiple comparisons
☐	☒	A full description of the statistical parameters including central tendency (e.g. means) or other basic estimates (e.g. regression coefficient) AND variation (e.g. standard deviation) or associated estimates of uncertainty (e.g. confidence intervals)
☐	☒	For null hypothesis testing, the test statistic (e.g. *F*, *t*, *r*) with confidence intervals, effect sizes, degrees of freedom and *P* value noted<br>*Give P values as exact values whenever suitable.*
☒	☐	For Bayesian analysis, information on the choice of priors and Markov chain Monte Carlo settings
☒	☐	For hierarchical and complex designs, identification of the appropriate level for tests and full reporting of outcomes
☒	☐	Estimates of effect sizes (e.g. Cohen's *d*, Pearson's *r*), indicating how they were calculated

Our web collection on statistics for biologists contains articles on many of the points above.

Software and code

Policy information about availability of computer code

Data collection	The data collection methods were described in the Methods section. No custom software was used for data collection.<br>FACS: BD LSRFortessa; SONY Cell Sorter software v2.1.6<br>CFU: KEYENCE BZ Series v01.01.01.03<br>Mito stress: Seahorse XF Pro Analyzer<br>WB: CLARIOstar: v5.70 R3; Compass for SW.<br>qPCR: QuantStudio6Pro Design& analysis software v2.6.0<br>IF: Olympus FV3000R; ZEISS ZEN v2.3

Data analysis	All packages and programs used are available online. Custom code was not generated outside of the use of the predesigned packages.<br>SnapGene v6.0<br>Flowjo v10.7.1<br>ImageJ v2.1.0<br>TrimGalore v0.6.7<br>Hisat2 v2.1.0<br>FeatureCounts v2.0.1<br>ClusterProfiler package v4.2.2<br>Alpha fold v3<br>Pymol v3.1.0<br>MAGeCK v0.5.9

For manuscripts utilizing custom algorithms or software that are central to the research but not yet described in published literature, software must be made available to editors and reviewers. We strongly encourage code deposition in a community repository (e.g. GitHub). See the Nature Portfolio guidelines for submitting code & software for further information.

Data

Policy information about availability of data

All manuscripts must include a data availability statement. This statement should provide the following information, where applicable:

- Accession codes, unique identifiers, or web links for publicly available datasets
- A description of any restrictions on data availability
- For clinical datasets or third party data, please ensure that the statement adheres to our policy

Data have been deposited to GEO under the accession number GSE275462.

Research involving human participants, their data, or biological material

Policy information about studies with human participants or human data. See also policy information about sex, gender (identity/presentation), and sexual orientation and race, ethnicity and racism.

Reporting on sex and gender	N/A
Reporting on race, ethnicity, or other socially relevant groupings	N/A
Population characteristics	N/A
Recruitment	N/A
Ethics oversight	N/A

Note that full information on the approval of the study protocol must also be provided in the manuscript.

Field-specific reporting

Please select the one below that is the best fit for your research. If you are not sure, read the appropriate sections before making your selection.

☒ Life sciences ☐ Behavioural & social sciences ☐ Ecological, evolutionary & environmental sciences

For a reference copy of the document with all sections, see nature.com/documents/nr-reporting-summary-flat.pdf

Life sciences study design

All studies must disclose on these points even when the disclosure is negative.

Sample size	For all studies, experiments and sequencing were performed with biological replicates based on the standard in the field (PMID:33848471; PMID:28241143; PMID:38538791). Sample size for each experiment is indicated in the figure legends.
Data exclusions	Mice with transplantation failures were excluded from the study.
Replication	Reproducibility of findings were assessed via statistical tests, varying on sample size and experiment type. All the RNA-seq were repeated 3-4 times, and other standard assays (FACS, qPCR, IF, WB, Seahorse, co-IP, behavior test) were performed at least 3 independent times. We include in the figure legend all details relative to the sample size. We include in the methods all details relative to reagents and softwares. For all experiments, all attempts at replication were successful.
Randomization	Animals allocated to experimental groups were based on genotype and age. No method of randomization was used to assign mice to experimental groups.

Blinding

Group allocation for initial data collection was not performed blind to the investigators, but the group identities during data analysis were blinded to the investigators.

Reporting for specific materials, systems and methods

We require information from authors about some types of materials, experimental systems and methods used in many studies. Here, indicate whether each material, system or method listed is relevant to your study. If you are not sure if a list item applies to your research, read the appropriate section before selecting a response.

Materials & experimental systems

n/a	Involved in the study
☐	☒ Antibodies
☐	☒ Eukaryotic cell lines
☒	☐ Palaeontology and archaeology
☐	☒ Animals and other organisms
☒	☐ Clinical data
☒	☐ Dual use research of concern
☒	☐ Plants

Methods

n/a	Involved in the study
☒	☐ ChIP-seq
☐	☒ Flow cytometry
☒	☐ MRI-based neuroimaging

Antibodies

Antibodies used

CD11b Pacific Blue BioLegend #101223 M1/70 1:100
CD45.2 FITC Thermo Fisher #11-0454-81 104 1:300
CD3 APC Thermo Fisher #17-0032-82 17A2 1:200
B220 PerCP-Cy5.5 Thermo Fisher #45-0452-80 RA3-6B2 1:200
CD45.1 APC-Cy7 BioLegend #110716 A20 1:200
cKit Pacific Blue BioLegend #105820 2B8 1:100
Sca1 APC BioLegend #108111 D7 1:200
CD48 PerCP-Cy5.5 BioLegend #103421 HM48-1 1:200
CD150 PE-Cy7 BioLegend #115913 TC15-12F12.2 1:200
CD127 BV421 BioLegend #135023 A7R34 1:100
Sca1 488 BioLegend #108115 D7 1:200
CD135 PE BioLegend #135305 A2F10 1:200
cKit APC BioLegend #105811 2B8 1:200
CD41 BV421 BioLegend #133911 MWReg30 1:100
CD11b (Mac-1) APC-Cy7 BioLegend #101225 M1/70 1:200
CD16/32 (FcR) PE-Cy7 BioLegend #101317 93 1:200
CD135 BV421 BioLegend #135313 A2F10 1:100
cKit BV605 BioLegend #105847 2B8 1:100
CD4 PE-Cy7 BioLegend #100421 GK1.5 1:200
CD8 BV605 BioLegend #100743 53-6.7 1:200
Ki67 APC-Cy7 Thermo Fisher #47-5698-80 SolA15 1:200
CD93 PE-Cy7 BioLegend #136505 AA4.1 1:200
IgM APC BioLegend #406509 RMM-1 1:200
IgD Pacific Blue BioLegend #405725 11-26c.2a 1:100
CD21/CD35 Pacific Blue BioLegend #123421 7 E9 1:100
CD23 BV605 BioLegend #101637 B3B4 1:100
CD44 APC BioLegend #103011 IM7 1:200
CD62L Pacific Blue BioLegend #104435 MEL-14 1:100
Tom20 Santa sc-17764 1:200
ApoJ (Clu) Thermo PA5-46931 1:2000
p-p38 CST 9216S 1:2000
v5 tag Abcam ab15828 1:2000
Mfn2 CST 9482T 1:1500
Mfn1 CST 13798-1-AP 1:1500
Drp1 CST 8570T 1:1500
Myc tag CST 2278T 1:3000
Tomm20 Abcam AB56783 1:300
Clu Santa sc-5289 1:1000
p-p38 BD 612288 1:2000
V5 tag Thermo R960-25 1:2000
Mfn2 Abcam AB56889 1:2000
Mfn1 Thermo 66776-1-IG 1:1500
Dlp1 BD 611112 1:2000
Myc tag Thermo MA1-21316 1:2000
53BP1 Bio-techne NB100-904 1:300
β-Actin CST 3700S 1:3000
Lamp2a Abcam ab18528 1:200

	anti-Mouse-HRP Thermo SA1-100 1:1000<br>anti-Rabbit-HRP Thermo 31458 1:5000<br>anti-Goat_HRP Thermo A16005 1:2000
Validation	https://www.biolegend.com/fr-lu/products/pacific-blue-anti-mouse-human-cd11b-antibody-3863<br>https://www.thermofisher.com/antibody/product/CD45-2-Antibody-clone-104-Monoclonal/11-0454-82<br>https://www.thermofisher.com/antibody/product/CD3-Antibody-clone-17A2-Monoclonal/17-0032-82<br>https://www.thermofisher.com/antibody/product/CD45R-B220-Antibody-clone-RA3-6B2-Monoclonal/45-0452-82<br>https://www.biolegend.com/fr-lu/products/apc-cyanine7-anti-mouse-cd45-1-antibody-2320<br>https://www.biolegend.com/fr-lu/products/pacific-blue-anti-mouse-cd117-c-kit-antibody-3133<br>https://www.biolegend.com/fr-lu/products/apc-anti-mouse-ly-6a-e-sca-1-antibody-225<br>https://www.biolegend.com/fr-lu/products/percp-cyanine5-5-anti-mouse-cd48-antibody-5597<br>https://www.biolegend.com/fr-lu/products/pe-cyanine7-anti-mouse-cd150-slam-antibody-3056<br>https://www.biolegend.com/fr-lu/products/brilliant-violet-421-anti-mouse-cd127-il-7ralpha-antibody-7193<br>https://www.biolegend.com/fr-lu/products/alexa-fluor-488-anti-mouse-ly-6a-e-sca-1-antibody-3138<br>https://www.biolegend.com/fr-lu/products/pe-anti-mouse-cd135-antibody-6173<br>https://www.biolegend.com/fr-lu/products/apc-anti-mouse-cd117-c-kit-antibody-72<br>https://www.biolegend.com/fr-lu/products/brilliant-violet-421-anti-mouse-cd41-antibody-7558<br>https://www.biolegend.com/fr-lu/products/apc-cyanine7-anti-mouse-human-cd11b-antibody-3930<br>https://www.biolegend.com/fr-lu/products/pe-cyanine7-anti-mouse-cd16-32-antibody-6355<br>https://www.biolegend.com/fr-lu/products/brilliant-violet-421-anti-mouse-cd135-antibody-8728<br>https://www.biolegend.com/fr-lu/products/brilliant-violet-605-anti-mouse-cd117-c-kit-antibody-16969<br>https://www.biolegend.com/fr-lu/products/pe-cyanine7-anti-mouse-cd4-antibody-1919<br>https://www.biolegend.com/fr-lu/products/brilliant-violet-605-anti-mouse-cd8a-antibody-7636<br>https://www.thermofisher.com/antibody/product/Ki-67-Antibody-clone-SolA15-Monoclonal/47-5698-82<br>https://www.biolegend.com/fr-lu/products/pe-cyanine7-anti-mouse-cd93-aa4-1-early-b-lineage-antibody-6420<br>https://www.biolegend.com/fr-lu/products/apc-anti-mouse-igm-2335<br>https://www.biolegend.com/fr-lu/products/brilliant-violet-421-anti-mouse-igd-9033<br>https://www.biolegend.com/fr-lu/products/brilliant-violet-421-anti-mouse-cd21-cd35-cr2-cr1-antibody-7557<br>https://www.biolegend.com/fr-lu/products/brilliant-violet-605-anti-mouse-cd23-antibody-18905<br>https://www.biolegend.com/fr-lu/products/apc-anti-mouse-human-cd44-antibody-312<br>https://www.biolegend.com/fr-lu/products/brilliant-violet-421-anti-mouse-cd62l-antibody-7164<br>https://www.scbt.com/p/tom20-antibody-f-10?srsltid=AfmBOopHNph9xlF5bgXwrkmQktBQp-tI95uD1tZ0rU25ue4-px5J6zGG<br>https://www.thermofisher.com/antibody/product/Apolipoprotein-J-Antibody-Polyclonal/PA5-46931<br>https://www.cellsignal.com/products/primary-antibodies/phospho-p38-mapk-thr180-tyr182-28b10-mouse-mab/9216?<br>srsltid=AfmBOoorXItaT_UoCmT6YY3vG73oDzZvyRNU6FtVq6Gpk1fTTXxCZQwW<br>https://www.abcam.com/en-us/products/primary-antibodies/v5-tag-antibody-ab15828?srsltid=AfmBOorZz_aRvi22bwkNg-<br>BqeDLnqKzxIdu5u8MlSEUF2zA6zKBRNQyD<br>https://www.cellsignal.com/products/primary-antibodies/mitofusin-2-d2d10-rabbit-mab/9482?<br>srsltid=AfmBOoqUSUv05WmCJNCj9_l8JtB0IH-qOqHIIXC31pBltVFmbc2eoX5x<br>https://www.ptglab.com/products/MFN1-Antibody-13798-1-AP.htm?srsltid=AfmBOooDAbw5_1z1tkk1yzq4QWqTnNesQJLIe-<br>BJrAZndoSjKXj12rLu#product-information<br>https://www.cellsignal.com/products/primary-antibodies/drp1-d6c7-rabbit-mab/8570?<br>srsltid=AfmBOooDFaJR07f09EtN3ZJQDcvgfwHNddwVk1ewCe47LNktr1Vxt2f4<br>https://www.cellsignal.com/products/primary-antibodies/myc-tag-71d10-rabbit-mab/2278?<br>srsltid=AfmBOorQ9ZIr76DKITo42GwNZ_4xPVDM_WjzUrgbZoUvVbjQ487WCKNj<br>https://www.abcam.com/en-us/products/primary-antibodies/tomm20-antibody-4f3-bsa-and-azide-free-ab56783?<br>srsltid=AfmBOopmr4alYu77yMfVtMcPVEf3IxUGwlEhTpifLFdTnHLX4XhEvERp<br>https://www.scbt.com/p/clusterin-alpha-antibody-b-5?srsltid=AfmBOop1rfjaxCxkdX0hhRTgKtKzPCazfaiTuP_YUB-NSjFT4ZaWC6aL<br>https://www.bdbiosciences.com/en-eu/products/reagents/flow-cytometry-reagents/research-reagents/single-color-antibodies-ruo/<br>purified-mouse-anti-p38-mapk-pt180-py182.612288?tab=product_details<br>https://www.thermofisher.com/antibody/product/V5-Tag-Antibody-clone-SV5-Pk1-Monoclonal/R960-25<br>https://www.abcam.com/en-us/products/primary-antibodies/mitofusin-2-antibody-6a8-ab56889?<br>srsltid=AfmBOoq6yDB1AfAUwJgUVzZAGjipAy69p4x6ydO9U78CX_FC77u4mKXt<br>https://www.thermofisher.com/antibody/product/MFN1-Antibody-clone-3F11C11-Monoclonal/66776-1-IG<br>https://www.bdbiosciences.com/en-eu/products/reagents/microscopy-imaging-reagents/immunofluorescence-reagents/purified-<br>mouse-anti-dlp1.611112?tab=product_details<br>https://www.thermofisher.com/antibody/product/Myc-Tag-Antibody-clone-Myc-A7-Monoclonal/MA1-21316<br>https://www.novusbio.com/products/53bp1-antibody_nb100-904?<br>srsltid=AfmBOopo_J_GsBBkHYiNTUivv1OSKGG6JRitEgmhhPwy3EQP9E324Uk3<br>https://www.cellsignal.com/products/primary-antibodies/b-actin-8h10d10-mouse-mab/3700?srsltid=AfmBOopdmUB-<br>QNjAekrn0vmdd131p7VdADGAb8SoXbed3SYDi8Ehjm5X<br>https://www.abcam.com/en-us/products/primary-antibodies/lamp2a-antibody-lysosome-marker-ab18528?srsltid=AfmBOor-<br>eJDcf8jsZiQwCrwDnmFmRwdxT_67C7_gu7Nekdb8nrHxeMmW

Eukaryotic cell lines

Policy information about cell lines and Sex and Gender in Research

Cell line source(s)	HEK293T cells were purchased from ATCC (catologue # CRL-3216). WT MEFs (SCRC-1008), Mfn1-null MEFs (CRL-2992) and Mfn2-null MEFs (CRL-2993) were purchased from ATCC. HPC-7 cell line was kindly provided by Dr. Xuan Pan.
Authentication	The cell lines have been validated by ATCC. Cell line authentication was performed by ATCC using morphology and STR profiling. HPC-7 cell line authentication was performed by morphology and transcriptome.

Mycoplasma contamination	All the cell lines were tested negative for mycoplasma contamination.
Commonly misidentified lines (See ICLAC register)	No commonly misidentified lines were used in this study.

Animals and other research organisms

Policy information about studies involving animals; ARRIVE guidelines recommended for reporting animal research, and Sex and Gender in Research

Laboratory animals	The 8-10 weeks Cas9 mice were purchased from Jackson lab (#026179). The 8-10 weeks CD45.1 mice were purchased from Jackson lab (#002014). The 8-10 weeks young and 90 weeks old C57BL/6J mice was purchased from Jackson lab (#000664). The aged Cas9 mice (>20 months) was a gift from the Feng Zhang Lab at Broad Institute. All mice were kept under specific pathogen-free conditions within an environment controlled for temperature (20-22°C) and humidity (40-70%), and were subjected to a 12-hour light/dark cycle.
Wild animals	No wild animals were used in the study.
Reporting on sex	Male or female Cas9 mice were used for HSCs isolation. Male CD45.1 mice were used as transplantation recipient mice and for behavioral testing.
Field-collected samples	This study did not involve field-collected samples.
Ethics oversight	All animal experiments were performed in accordance with the protocols of the Institutional Animal Care and Use Committee at Harvard Medical School and Boston Children's Hospital.

Note that full information on the approval of the study protocol must also be provided in the manuscript.

Plants

Seed stocks	N/A
Novel plant genotypes	N/A
Authentication	N/A

Flow Cytometry

Plots

Confirm that:

☒ The axis labels state the marker and fluorochrome used (e.g. CD4-FITC).

☒ The axis scales are clearly visible. Include numbers along axes only for bottom left plot of group (a 'group' is an analysis of identical markers).

☒ All plots are contour plots with outliers or pseudocolor plots.

☒ A numerical value for number of cells or percentage (with statistics) is provided.

Methodology

Sample preparation	The humerus, pelvic, femur, and tibia bones of the donor mice were isolated. Bone marrow was extracted by cutting and crushing via scissors in cell culture dishes containing 1 × PBS. A serological pipette was used to repeatedly flush the bone marrow from the bones until all bone marrow was removed, indicated by visibly white bone material. Following filtering, the cells were centrifuged at 400g for 10 minutes and resuspended with PBS in a FACS tube. Donor HSCs were then isolated via magnetic separation using Streptavidin RapidSpheres™ from the EasySep™ Mouse Hematopoietic Progenitor Cell Isolation Kit (StemCell Technologies, # 19856) according to the manufacturer's instructions. After HSPC isolation and the removal of lineage positive cells, the remaining cells were centrifuged at 400g and resuspended with PBS. For FACS sorting, the cells were stained with antibodies for 20 min at 4°C and washed twice with PBS. Cells were sorted with SH800S Cell Sorter (Sony Biotechnology) into 1.5 ml tubes and stored on ice for further use.<br><br>Peripheral blood was collected from the recipient mice in 1.5 ml tubes containing of EDTA. Red Blood Cell Lysis Buffer (Invitrogen, cat #00-4333-57) was added to lyse the red blood cells. The tubes were incubated at RT for 10 min on a rotator. 1 × PBS was added to the tubes and centrifuged at 400g for 5 min at RT to wash the cells. The tubes were washed and

	centrifuged again with PBS, and supernatants were discarded. Cell pellets were resuspended with 100 µl of PBS for antibody staining, and peripheral blood analysis was performed using LSR Fortessa (BD Biosciences).
Instrument	BD LSRFortessa and SONY cell sorter SH800Z.
Software	FlowJo v10.7.1
Cell population abundance	A minimum of 100,000 cells per sample were analyzed.
Gating strategy	Gating strategy was based on FSC-A, SSC-A to separate single cells and exclude any doublets by FSC-A/FSC-H profile. Gating strategies for all cell populations as described in several publications (PMID:27111842; PMID: 26095048; PMID: 34459852; PMID: 38538791).

☒ Tick this box to confirm that a figure exemplifying the gating strategy is provided in the Supplementary Information.
